# Distinct Phenotypes Caused by Mutation of *MSH2* in Trypanosome Insect and Mammalian Life Cycle Forms Are Associated with Parasite Adaptation to Oxidative Stress

**DOI:** 10.1371/journal.pntd.0003870

**Published:** 2015-06-17

**Authors:** Viviane Grazielle-Silva, Tehseen Fatima Zeb, Jason Bolderson, Priscila C. Campos, Julia B. Miranda, Ceres L. Alves, Carlos R. Machado, Richard McCulloch, Santuza M. R. Teixeira

**Affiliations:** 1 Departamento de Bioquímica e Imunologia, Universidade Federal de Minas Gerais, Belo Horizonte, Minas Gerais, Brazil; 2 The Wellcome Trust Center for Molecular Parasitology, Institute of Infection, Immunity and Inflammation, University of Glasgow, Glasgow, Scotland, United Kingdom; Liverpool School of Tropical Medicine, UNITED KINGDOM

## Abstract

**Background:**

DNA repair mechanisms are crucial for maintenance of the genome in all organisms, including parasites where successful infection is dependent both on genomic stability and sequence variation. MSH2 is an early acting, central component of the Mismatch Repair (MMR) pathway, which is responsible for the recognition and correction of base mismatches that occur during DNA replication and recombination. In addition, recent evidence suggests that MSH2 might also play an important, but poorly understood, role in responding to oxidative damage in both African and American trypanosomes.

**Methodology/Principal Findings:**

To investigate the involvement of MMR in the oxidative stress response, null mutants of *MSH2* were generated in *Trypanosoma brucei* procyclic forms and in *Trypanosoma cruzi* epimastigote forms. Unexpectedly, the *MSH2* null mutants showed increased resistance to H_2_O_2_ exposure when compared with wild type cells, a phenotype distinct from the previously observed increased sensitivity of *T*. *brucei* bloodstream forms *MSH2* mutants. Complementation studies indicated that the increased oxidative resistance of procyclic *T*. *brucei* was due to adaptation to MSH2 loss. In both parasites, loss of MSH2 was shown to result in increased tolerance to alkylation by MNNG and increased accumulation of 8-oxo-guanine in the nuclear and mitochondrial genomes, indicating impaired MMR. In *T*. *cruzi*, loss of MSH2 also increases the parasite capacity to survive within host macrophages.

**Conclusions/Significance:**

Taken together, these results indicate MSH2 displays conserved, dual roles in MMR and in the response to oxidative stress. Loss of the latter function results in life cycle dependent differences in phenotypic outcomes in *T*. *brucei MSH2* mutants, most likely because of the greater burden of oxidative stress in the insect stage of the parasite.

## Author Summary

*Trypanosoma brucei* and *Trypanosoma cruzi* are protozoa parasites that cause sleeping sickness and Chagas disease, respectively, two neglected tropical diseases endemic in sub-Saharan Africa and Latin America. The high genetic diversity found in the *T*. *cruzi* population and the highly diverse repertoire of surface glycoprotein genes found in *T*. *brucei* are crucial factors that ensure a successful infection in their hosts. Besides responding to host immune responses, these parasites must deal with various sources of oxidative stress that can cause DNA damage. Thus, by determining the right balance between genomic stability and genetic variation, DNA repair pathways have a big impact in the ability of these parasites to maintain infection. This study is focused on the role of a DNA mismatch repair (MMR) protein named MSH2 in protecting these parasites’ DNA against oxidative assault. Using knock-out mutants, we showed that, besides acting in the MMR pathway as a key protein that recognizes and repairs base mismatches, insertions or deletions that can occur after DNA replication, MSH2 has an additional role in the oxidative stress response. Importantly, this extra role of MSH2 seems to be independent of other MMR components and dependent on the parasite developmental stage.

## Introduction

Two members of the trypanosomatidae family, *Trypanosoma cruzi* and *Trypanosoma brucei*, are important human pathogens, since they cause, respectively, Chagas disease, or American trypanosomiasis, and African Sleeping Sickness, or Human African trypanosomiasis. Together, *T*. *cruzi* and *T*. *brucei* infections affect almost 20 million people [1, 2]. The life cycles of both these parasites involve two hosts: an invertebrate vector and a mammalian host. In the digestive tract of the insect vector *T*. *cruzi* multiplies as epimastigotes and differentiates into metacyclic trypomastigotes, which are expelled with the vector’s faeces. After a blood meal, trypomastigotes injected in the host bloodstream can invade different cell types, where they replicate as intracellular amastigotes that, after a number of replication cycles in the host cell cytoplasm, differentiate into trypomastigotes and lyse the host cell membrane. Despite being similar in general strategy, the life cycle of *T*. *brucei* is different to that of *T*. *cruzi* in several key details. Notably, *T*. *brucei* does not display any intracellular replicative stages. In the mammal, *T*. *brucei* is exclusively extracellular, replicating in the bloodstream and tissue fluids as bloodstream form (BSF) cells, which can be taken up by the tsetse fly vector during a bloodmeal. In the insect vector BSF cells differentiate into replicative procyclic forms (PCF), which then undergo several further differentiation events associated with migration to the fly salivary glands, where non-replicative metacyclic trypomastigotes are formed and can be passed into a new mammalian host through the proboscis when the infected fly is feeding [3]. Irrespective of the detailed differences in the life cycles, differentiation between the mammal-infective and vector-infective forms of both *T*. *cruzi* and *T*. *brucei* is accompanied by dramatic metabolic changes and morphological alterations [4].

The ability to multiply and survive inside a host or vector is crucial for the maintenance of a parasite infection and transmission, allowing continuation of the life cycle. As for any cell, unicellular parasites are exposed to potentially deleterious events during cell division. The by-products of cellular metabolism, allied to routine errors during DNA replication or recombination processes, represent endogenous sources of potential DNA damage and genome change. In addition, all organisms are subjected to exogenous genotoxic agents from the environment or, in the case of parasites, derived from host. In the mammalian host, *T*. *cruzi* invades non-phagocytic cells or can be internalized by macrophages by a phagocytosis-like process [5]. Inside macrophages *T*. *cruzi* triggers the activation of NADPH oxidase, which generates large amounts of reactive oxygen species (ROS) such as O_2_⠁^-^. Moreover, pro-inflammatory cytokines triggered by *T*. *cruzi* infection also stimulate infected macrophages to produce high amounts of nitric oxide (⠁NO) through the induction of inducible nitric oxide synthase (iNOS), which can react with O_2_⠁^-^ producing peroxynitrite (ONOO^-^), a powerful oxidant and cytotoxic molecule [6, 7]. Similarly, the insect life forms of both parasites must also deal with the invertebrate oxidative stress response generated against the parasite. Upon challenge with *T*. *brucei* the tsetse fly activates iNOS, generating ⠁NO, and increases the levels of ROS such as hydrogen peroxide (H_2_O_2_) [8, 9]. To deal with all of these potentially genome damaging agents, trypanosomes, like any other organism, possess multiple DNA repair pathways [10, 11], though little work has detailed to what extent these pathways are needed in different parasite life cycle stages. One such DNA repair pathway is Mismatch Repair (MMR), which acts to detect and correct mispaired bases that escape the proofreading activity of DNA polymerases during replication or occur during recombination between non-identical DNA molecules. In eukaryotic cells, even small distortions in DNA caused by single-base mismatches or insertion/ deletion loops (IDLs) of 1–3 bases are recognised by the “sliding clamp” heterodimer formed by MSH2 and MSH6 proteins, which moves along DNA searching for errors [12]. Larger IDLs are recognised by a distinct heterodimer, composed of MSH2 and MSH3. Eukaryotes encode several further MSH proteins, all of which are homologous to bacterial MutS proteins, though many have adopted non-MMR roles [13]. Following mismatch recognition, an ATP-dependent conformation change in MSH2-6 or MSH2-3 promotes interaction with heterodimeric homologues of bacterial MutL proteins, bringing these factors to the site of the lesion. The excision step is catalyzed by an endonuclease activity within the MutL-related heterodimer formed between MLH1-PMS1 [14], and that activity is activated by PCNA (a component of replication machinery) [15] [16]. A defect in or directed inactivation of the MMR pathway leads to a 100–1000 fold increase in spontaneous mutation rate in all organisms examined, including *Escherichia coli* [17], *Saccharomyces cerevisiae* [18], *Caenorhabditis elegans* [19, 20], and *T*. *brucei* [21]. In human cells and murine models MMR deficiency is correlated with predisposition to cancer [22, 23]. MMR suppresses mutations by increasing DNA replication fidelity through preventing base substitutions or repeat sequence instability, events that can also occur during homologous recombination [24].

In addition to repair of non-identical DNA sequences, MMR has also been demonstrated to be involved in the response to DNA damage induced by genotoxic agents, among them the alkylator N-methyl-N’-nitro-N-nitrosoguanidine (MNNG), the anti-cancer drug cisplatin and H_2_O_2_ [25]. Methylation of DNA by MNNG gives rise to *O* ^*6*^-methylguanine (*O* ^*6*^-MeG), opposite which thymine can be misincorporated during replication, forming a base mismatch recognised by MMR. Defective MMR is associated with increased tolerance to MMNG [26], due to loss of what has been termed a futile cycle of repair, where MMR fails to remove *O* ^*6*^-MeG and instead re-inserts either cytosine or thymine opposite the lesion. In some organisms, active MMR at MNNG damage leads to persistent DNA breaks and increased cell death [27]. H_2_O_2_ generates reactive oxygen species (ROS) with high capacity to damage DNA [28]. The most common DNA lesion resulting from ROS is 7,8-dihydro-8-oxoguanine (8-oxoG) [29]. MMR has the ability to prevent oxidative mutagenesis in *E*. *coli* by removing 8-oxoG or adenine misincorporated paired with 8-oxoG [30]. In *S*. *cerevisiae* it was demonstrated that the MSH2-MSH6 heterodimer has a direct role in removing adenine in 8-oxoG:A mispaired bases [31]. More recently, Zlatanou *et al*. proposed a non-MMR role for MSH2-MSH6 in human fibroblasts in response to oxidative damage. The model suggests that in some circumstances DNA oxidative damage by MSH2-MSH6 does not recruit other MMR proteins, but instead repair is mediated through the action of monoubiquinated-PCNA (mUb-PCNA) and Polη [32].

With the availability of the complete genome sequences of *T*. *brucei* and *T*. *cruzi* the full repertoire of the parasite’s putative DNA MMR machinery was revealed to comprise homologues of MSH2, MSH3, MSH6 (originally named MSH8) [21], MLH1 and PMS1 [10, 33, 34]. However, functional characterization studies to date have been limited to a single life cycle stage in each parasite and have only tested the roles of MSH2 in *T*. *brucei* and *T*. *cruzi*, and MLH1 in *T*. *brucei*. Null mutants of *T*. *brucei msh2* (*Tbmsh2-/-*) or *mlh1* (*Tbmlh1-/-*) in BSF cells show several phenotypes characteristic of MMR-deficient cell lines: increased tolerance to MNNG, increased rates of sequence change in a number of microsatellite repeat loci, and elevated rates of homologous recombination-based integration of transformed DNA molecules carrying base mismatches relative to genomic target loci [21, 35, 36]. In addition, evidence that TbMSH2 and TcMSH2 play a role in in the oxidative stress response has been found in studies showing that *Tbmsh2-/-* BSF cells and *Tcmsh2+/-* epimastigotes are more susceptible to H_2_O_2_ treatment than wild type parasites [21, 37]. The role played by MSH2 in tackling oxidative damage appears not to involve a complete MMR reaction, since BSF *Tbmlh1-/-* mutants do not display equivalent increased H_2_O_2_ sensitivity to *Tbmsh2*-/- mutants [38]. Moreover, heterologous expression of *T*. *cruzi* MSH2 in *T*. *brucei msh2*-/- mutants shows that the American trypanosome MSH2 protein can functionally replace the endogenous MSH2 protein in the oxidative stress response, but cannot work with the *T*. *brucei* MMR machinery to successfully execute MMR [38]. Although only single allele knock-outs of *msh2* have been described in *T*. *cruzi* epimastigotes, the *T*. *cruzi* msh2+/- mutants display increased sensitivity to H_2_O_2_ treatment [37], consistent with the null mutants in *T*. *brucei* BSF cells [38]. However, despite evidence in both *T*. *brucei* and *T*. *cruzi* that H_2_O_2_-induced damage predominantly affects the kinetoplast genome when MSH2 is lost or impaired [37], the exact role of MSH2 remains elusive. Here, we have sought to clarify the role of MSH2 in the oxidative stress response of *T*. *brucei* and *T*. *cruzi*, and to ask to what extent this putative MMR-related function is conserved or diverged in the two parasites. We describe the generation of *msh2* null mutants in *T*. *brucei* PCF cells, which differ from BSF cells in the increased use of mitochondrial metabolism [39], with the consequence that there is greater endogenous reactive oxygen species. In addition, we describe, for the first time, the generation of *msh2* null mutants in *T*. *cruzi* epimastigote cells, which also rely on aerobic metabolism [40]. In both cell types, *msh2* null mutants are viable and are impaired in MMR. Unexpectedly, both mutants do not display increased sensitivity to H_2_O_2_ treatment, but instead, increased resistance, which we suggest to be due to parasite adaptation to the loss of a crucial molecule. Thus, we propose that MSH2 provides one of several interconnected mechanisms that are common to *T*. *brucei* and *T*. *cruzi* and allow both parasites to cope with oxidative stress.

## Methods

### Parasite cultures

*T*. *brucei* cultures of the Lister 427 strain were maintained as both bloodstream (BSF) and procyclic (PCF) forms. BSF parasites were maintained at 37°C, 5% CO_2_ in HMI-9 (GIBCO) medium supplemented with 20% fetal bovine serum (GIBCO). Cell passages were performed every 48 hours, with population density maintained between 1 x 10^5^ and 2 x 10^6^ cells.mL^-1^. PCF cells were maintained at 27°C in SDM-79 (GIBCO) medium supplemented with 10% fetal bovine serum (GIBCO). Weekly passages were performed, but cell density was never lower than than 5 x 10^5^ cells.mL^-1^.

Epimastigote forms of the CL Brener clone of *T*. *cruzi* were maintained in logarithmic growth phase at 28°C in liver infusion tryptose (LIT) medium supplemented with 10% fetal bovine serum (GIBCO) and penicillin (10,000 U.mL^-1^)/Streptomycin (10,000 μg.mL^-1^) (GIBCO) as described by [41]. Metacyclic trypomastigotes, obtained after metacyclogenesis of epimastigotes cultures maintained in LIT medium for 15 to 20 days, were used to infect Vero cells cultured in DMEM medium (GIBCO) supplemented with 5% fetal bovine serum (GIBCO).

### Parasite transfections

Transfection of PCF parasites were carried out at a density of 5 x10^6^ cells.mL^-1^ resuspended in 0.5 mL of ice-cold Zimmerman medium (132 mM NaCl, 8 mM KCl, 8 mM Na_2_HPO_4_, 1.5 mM KH_2_PO_4_, 0.5 mM MgAc_2_, and 0.06 mM CaAc_2_, pH 7.5) and 10 μg of DNA. The mixture was subjected to two rounds of electroporation with a Bio-Rad Gene Pulser II (1.5 kV current and 25 μF capacitance). The cells were then transferred into SDM79 medium and incubated at 27°C overnight. To select for antibiotic-resistant transfectants, parasites were diluted 1:100 and 1:10 in 96 well plates containing conditioned medium and the appropriate antibiotic and incubated for 10–14 days.

Transfection of BSF cells were carried out using AMAXA Nucleofactor (Amaxa Biosystems) with 4 x 10^7^ cells resuspended in 100 μL of nucleofector solution (optimised for human T-cells) and mixed with 5–10 μg of DNA. After following the nucleofection protocol according to manufacturer instructions, the cells were serially diluted 1:10, 1:100 and 1:1000 in HMI-9 medium without antibiotic and incubated 6–12 hrs for recovery, after which 1 mL of HMI-9 medium containing antibiotic was added to each well. To select for *MSH2* or *MLH1* single allele mutants, PCF cells were selected with either 10 μg.mL^-1^ blasticidin or 1 μg.mL^-1^ puromycin; both drugs, at the same concentrations, were used to select for deletion of the second allele in the single allele mutants. To select for PCF *msh2*-/- cells in which MSH2 was re-expressed, transformants were grown in the presence of 2.5 μg.mL^-1^ phleomycin.

Transfection of *T*. *cruzi* epimastigotes was carried out with 10^7^ cells.mL^-1^ collected during exponential growth phase and 100 μg of plasmid constructions as previously described [42]. Twenty-four hours after transfecting hygromycin resistant cell lines, 100 μg.mL^-1^ of selective antibiotic (G-418 Sulfate) was added to transfected cultures. Weekly passages were performed with increasing concentration of the selective drug (up to 200 μg.mL^-1^). After 30–40 days, a double-resistant population was selected and cloned cell lines were obtained by plating epimastigotes on semisolid blood agarose plates with 200 μg.mL^-1^ of G-418 Sulfate and Hygromycin B, after an additional 30 days of incubation at 28°C.

### Plasmid constructs to generate knockout parasites, MSH2 re-expressers and to inhibit msh2 expression by RNAi

Knockout constructs to delete *msh2* (TritrypDB Tb927.10.11020) or *mlh1* (TritrypDB Tb927.8.6840) in *T*. *brucei* PCF cells have been described before [21] and were generated by PCR-amplifying the 5’ and 3’ UTRs corresponding to each gene from TREU 927 genomic DNA and cloning into pBluescript II KS and separated by antibiotic resistance gene (BSD and/or PUR), flanked by 230 bp of β-α tubulin and 330 bp of α-β tubulin processing signals. The constructs were linearized by digestion with *Xho*I and *Not*I restriction enzymes before transfecting into *T*. *brucei* PCF.

Epimastigote cultures of *T*. *cruzi* with one of the *msh2* alleles disrupted by a Hygromycin resistance gene was generated as described by [37]. The deletion of the second allele was performed using a DNA construct containing the Neomycin phosphotransferase gene (Neo) flanked by intergenic sequences of the *HX1* and *GAPDH* regions that were PCR-amplified from the pROCK-GFP vector [42] and cloned into pGEM T-easy vector (Promega). DNA sequence corresponding to ~500 bp of the 5’ and 3’ end of *TcMSH2* (TritrypDB TcCLB.507711.320) coding region was inserted upstream and downstream of *HX1* and *GAPDH*, respectively, in the pTopo_HX1_Neo_GAPDH and the plasmid linearized using the *Xba*I and *Sma*I restriction enzyme digestions before transfection.

*T*. *brucei msh2-/-* BSF and PCF were transfected with an MSH2 re-expression construct, which has been described before [21] and where the *msh2* ORF integrates and replaces either the *BSD* or *PUR* resistance cassette in the mutated *msh2-/-* locus. This construct was generated using a 4.5 kb region containing the *MSH2* gene flanked by *MSH2* 5’UTR and 3’UTR. The 4.5 kb fragment was cloned into pBluescript SK II, followed by a cloned phleomycin resistant cassette downstream of the 3’ UTR of the *MSH2* gene. The construct was digested with *Hind*III prior to transfection.

To generate RNAi constructs for MSH2, a 513bp region of the gene was PCR-amplified from wild type Lister 427 genomic DNA and cloned into the vector pZJM [43] where they are flanked by opposing T7 promoters and Tet operator sequences. The MSH2 RNAi plasmid was linearized with *Not*I and used to transform *T*. *brucei* PCF strain 427 pLew29-pLew13 developed by Wirtz *et al* [44], constitutively co-expressing T7 RNA polymerase and Tet repressor. Clones were selected with 2.5 μg.ml^-1^ phleomycin.

### RNA extraction, northern blot, cDNA generation, reverse transcriptase (RT) reaction and quantitative RT-PCR

Total RNA was isolated from *T*. *brucei* PCF form and *T*. *cruzi* epimastigote forms using the RNeasy kit (Qiagen). For cDNA generation RNA samples were treated, on column, with RNAse-free DNAse (Qiagen) as per the manufacturer’s instructions. cDNA was synthesized using High Capacity RNA-cDNA master mix (Applied Biosystems). cDNA from wild type (WT) cells, *Tbmsh2+/-* blasticidin (BSD) or puromycin (PUR) resistant clones, *Tbmsh2-/-*, *Tbmlh1+/-* BSD or PUR resistant clones and *Tbmlh1-/-* were used in reverse transcriptase (RT) PCR with specific primers for the *TbMSH2* or *TbMLH1* genes. As a positive control, primers to specifically PCR-amplify *TbRAD51* were used. For northern blot analyses, 20 μg of total RNA of *T*. *cruzi* WT, *Tcmsh2+/-* and three different clones of *Tcmsh2-/-* were separated in a 1.2% agarose/MOPS/formaldehyde gel. The RNA was then transferred to hybond-N+membrane (GE Healthcare) and hybridized with a *TcMSH2* specific PCR fragment previously labelled with [α-^32^P]-dCTP using the Amersham Ready-to-Go DNA Labelling Beads (GE Healthcare). The hybridization was carried out as previously described by [45]; briefly, hybridisation was in 50% formamide buffer overnight at 42°C, after which the blot was washed twice with 2× SSC/ 0.1% SDS at 65°C for 20 min. The membrane was then exposed to a phosphor screen of the STORM 820 phosphor image (GE Healthcare) and analysed by ImageQuant TL software (GE Healthcare). To quantify levels of mRNA by RT-PCR after RNAi induction, primers specific for *msh2* and *mlh1* genes were used. As a endogenous control primers for GPI8 gene (TritrypDB Tb427.10.13860) were used. SYBR Green PCR Master Mix (Applied Biosystems) was used for PCR in 96 well plates. Reactions were run on an ABI Prism 7000 thermocycler and mRNA levels quantified from amplification according to the manufacturer’s instructions;

### Microsatellite instability

*T*. *brucei* PCF MMR mutants were tested for genetic instability by PCR amplification of the region containing the JS-2 microsatellite from parasites grown in the absence or presence of 20 μM H_2_O_2_ for 48 hours. The JS-2 microsatellite has been mapped to Chromosome IV and is composed of GT-dinucleotide repeats [46]. Genomic DNA was extracted from 10 independent clonal populations derived from PCF wild type and knockout mutants and PCR-amplified with JS2 microsatellite complementary primers. PCR products were resolved for 50 minutes at 100 V by electrophoresis in a 3% low melting agarose gel.

### Treatment with genotoxic agents

Mid-log phase PCF *T*. *brucei* cultures were inoculated in SDM-79 (GIBCO) at density of 5 x 10^5^ cells.mL^-1^ in the absence or presence of 2.5 μM or 5 μM N-methyl-N’-nitro-N-nitrosoguanidine (MNNG, Tokyo chemical industry Ltd). After 72 hours survival was determined using a haematocytometer. *T*. *cruzi* epimastigote form cells in the exponential growth phase were counted and diluted to 1x 10^7^ cells.mL^-1^ in LIT medium in the absence or presence of 5 μM MNNG (Tokyo chemical industry Ltd). After 72 hours cell densities were determined by counting live cells with a haematocytometer using Erythrosin B exclusion.

Mid-log phase *T*. *brucei* PCF cells were diluted to 5 x 10^5^ cells.mL^-1^ in SDM-79 (GIBCO) and then incubated with 10 μM or 20 μM H_2_O_2_ (VWR) at 27°C for 48–72 hours. BSF cells were diluted to 1 x 10^6^ cells.mL^-1^ in HMI-9 medium (GIBCO) and incubated with 100 μM or 200 μM H_2_O_2_ (VWR) at 37°C, 5% CO_2_ for 48 hours. After growth, cell density was measured using a haematocytometer. *T*. *cruzi* epimastigote form in exponential growth phase were diluted to 1x 10^7^ parasites.mL^-1^ in PBS 1x and incubated with 75 μM H_2_O_2_ (Sigma-Aldrich) for 20 minutes at 28°C. After this, the cells were centrifuged, washed once with PBS and allowed to recover in LIT medium for 48 hours before cell densities were determined by counting live cells with a haematocytometer using Erythrosin B exclusion.

### Cell growth analyses after induction of RNAi

Two clones of PCF RNAi cell line targeting MSH2 were grown to log phase and diluted to a starting density of 5 x 10^5^ cells.mL^-1^. Each culture was then split and grown in the absence of tetracycline or after the addition of tetracycline to 2 μg.mL^-1^. Cell density was determined every 24 hours up to 72 hours and then the cultures were diluted to their starting density. After adding again tetracycline to induce siRNA expression, parasite numbers were further determined every 24 hours for a further 72 hours, before diluting again the cultures back to starting densities.

### *T*. *cruzi in vitro* infection of Vero cells and mouse macrophages

Trypomastigotes released in the supernatants of infected Vero cells were counted with a haemocytometer and an equivalent cell number for each culture was used to infect Vero cells or intraperitoneal macrophages (harvested from BALB/c mice) that had been allowed to adhere to glass cover slips in a 24 well plate. Trypomastigotes were incubated with the cell monolayers for 4 hours, after which non-internalized trypomastigotes were washed away with PBS 1x. The infected cells were incubated for an additional 48 hours at 37°C. The number of intracellular amastigotes was determined by staining cell nuclei with DAPI (1000 μg.mL^-1^) and visualised through fluorescence microscopy (Olympus). The average number of amastigotes was determined from analysing 1000 host cells, in different fields of the microscopy slide.

In vitro infection of BALB/c mice macrophages were carried out in strict accordance with the Brazilian laws regarding animal use (Law # 11.794, December 8, 2008), following a protocol approved by the Committee on the Ethics of Animal Experiments of UFMG (CETEA-UFMG) under the number 132/2014.

### 8-oxoguanine measurement

8-oxoG measurement was performed based on the principle of binding affinity of FITC-conjugated avidin to 8-oxoG [47] and an adapted protocol [48], which has been previously used to quantify oxidative DNA lesions in different *T*. *cruzi* strains and mutants for different DNA repair proteins [37, 49, 50]. Briefly, 20 μL aliquots of cell suspensions were distributed on a glass slide coated with poly-lysine (Sigma-Aldrich) and left to adhere for 5–10 min, after which cells were fixed with ice-cold methanol for 20 min at -20°C. The methanol-fixed cells were then washed with room temperature PBS 1x, cells permeabilized with 0.1% Triton X-100 for 3 min and incubated with RNase A (100 μg.mL^-1^) for 1 hour at 37°C. After another wash with PBS 1x, cells were treated with proteinase K (10 μg.mL^-1^) for 7 min at room temperature, followed by treatment with HCl (4N) for 7 min at room temperature and overnight blocking with 10% fetal bovine serum. FITC-avidin was diluted 1:200 in blocking buffer and incubated with the cells for 1 hour at room temperature. After extensive washing with PBS, cell nucleus and kDNA were stained with DAPI. Slides were mounted with prolong gold anti-fade solution (Molecular Probes/Life Technologies), visualized under a fluorescence microscope and images of at least 10 different fields were captured. After manually delimiting the nucleus and the kDNA of each parasite, as visualized by DAPI staining, FITC- fluorescence intensity restricted to these sites were determined with ImageJ software and plotted as the average fluorescence (arbitrary units) of 100 random cells.

### Epitope tagging MSH2

*T*. *brucei* BSF, PCF and *T*. *cruzi* epimastigote cells were transfected with constructs to tag MSH2 C-terminally in the endogenous locus. Primers were generated against a C-terminal region of *msh2* ORF, but excluding the stop codon. The region was selected to have a unique restriction enzyme recognition site within the ORF fragment that could be used to linearize the DNA. This fragment was cloned into the plasmid pNAT12^myc^ [51] allowing in frame fusion of the ORF with 12 repeats of the c-MYC epitope. Similarly, the C-terminal region of *Tcmsh2* ORF was cloned in pCR 2.1 Topo vector (Life Technologies) in frame with the 12 repeats of the c-MYC epitope derived from the *T*. *brucei* pNAT12^myc^ plasmid. As a selective mark, neomycin resistance gene derived from the pROCK_Neo plasmid [42] was also cloned in the same pCR 2.1 Topo vector. The constructs were linearized with *BmgB*I (for *T*. *brucei*) or *Sal*I (for *T*. *cruzi*) restriction enzymes and used to transfect heterozygous *msh2* knockout mutants of *T*. *brucei*, and wild type *T*. *cruzi* epimastigotes; transfected *T*. *brucei* or *T*. *cruzi* were selected with 10 μg.mL^-1^ blasticidin or 200 μg.mL^-1^ G418 Sulfate, respectively.

### Cellular localization

*T*. *brucei* BSF and PCF expressing MSH2 fused to 12x c-MYC epitopes were fixed in chilled methanol for 1 hour or overnight. Fixed slides were then blocked with 2% fetal calf serum (FCS), incubated with mouse anti-c-MYC antiserum (1:5000) (Millipore) for 1 hour, followed by incubation with Alexafluor 594 conjugated anti-mouse IgG secondary antiserum (Molecular Probes/ Life Technologies) in the dark. Slides were mounted with vectashield containing 4’,6-diamidino-2-phenylindole (DAPI; Vector labs) and visualized on a Zeiss Axioplan microscope. Images were captured using Hamamatsu ORCA-ER digital camera and Openlab software. *T*. *cruzi* epimastigote and amastigote cells expressing MSH2 fused to 12x c-MYC epitopes were fixed with 4% paraformaldehyde for 5 minutes, permeabilized with 0.1% Triton X-100 for 10 min, blocked with 1% BSA, 0.2% Tween 20 for 1 hour at room temperature and incubated with 1:500 anti-c-MYC antiserum conjugated to Alexa 488 (Milipore) for 1 hour. After washing with PBS, nuclei were stained with 1 μg.mL^-1^ of DAPI (Molecular Probes/ Life Technologies) for 5 min and cover slides mounted with prolong gold anti-fade solution (Molecular Probes/ Life Technologies). Images were captured on an Olympus BX60 microscope using Q-color 5 digital camera and Qcapture Pro 6.0 software.

### Statistical analysis

Statistical analyses in this work were performed using GraphPad Prism version 5.00 (GraphPad Software, San Diego California USA). Data are presented as mean plus standard deviation, and all experiments were repeated at least three times. Results were analysed for significant differences using ANOVA followed by Bonferroni post-test. Statistical tests used are described at each figure legend. The level of significance was set at P < 0.05.

## Results

### *T*. *brucei* procyclic form and *T*. *cruzi* epimastigote *msh2* null mutants have increased resistance to oxidative stress

**Fig 1:**
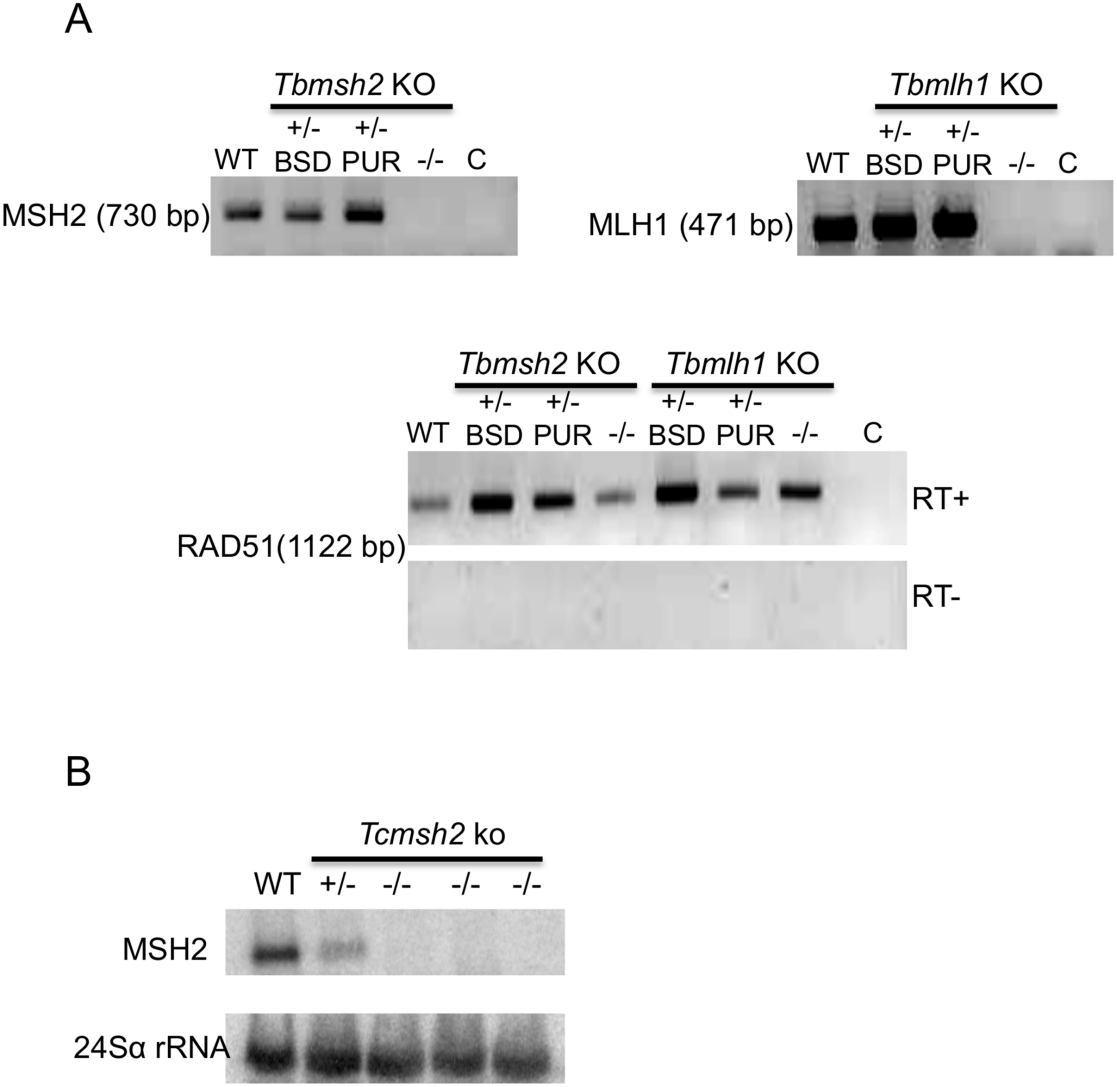
Generation of mismatch repair null mutants in *T*. *cruzi* and *T*. *brucei*. (**A**) Agarose gel electrophoresis of reverse transcriptase (RT) PCR products to verify the absence of *MSH2* or *MLH1* mRNA in *T*. *brucei* procyclic form mutants. cDNA derived from wild type (WT) cells, blasticidin (BSD) or puromycin (PUR) resistant clones of *Tbmsh2* single allele knockouts (+/-) and *Tbmsh2* double allele knockouts (-/-) were PCR-amplified with *MSH2* or *MLH1*- specific primers; the size of the PCR product is indicated and a control reaction without cDNA is indicated by C. The bottom panel shows, as positive controls, cDNAs from the same cells PCR-amplified (RT+) with primers specific for the *RAD51* gene; a control for genomic DNA contamination, in which reverse transcriptase was excluded from the cDNA synthesis reaction (indicated by RT-) is also shown. (**B**) Total RNA extracted from *T*. *cruzi* epimastigote form wild type (WT) cells, a *Tcmsh2* single allele knockout (+/-) mutant and three independent clones of *Tcmsh2* double allele knockouts (-/-) were transferred to a nylon membrane and hybridized with [α-^32^P]-labeled probe specific for the *T*. *cruzi MSH2* gene. The bottom panel shows hybridization with a probe for 24Sα rRNA, used as loading control.

The genomes of *T*. *brucei* and *T*. *cruzi* each contain a single-copy gene encoding MSH2 [33, 34]. To generate *T*. *brucei* PCF *msh2* null (-/-) mutants, we relied upon the same constructs used previously to make BSF cells in which both *msh2* alleles were deleted [21] (S1 Fig). In parallel, we also generated *T*. *brucei* PCF *mlh1*-/- mutants, again using pre-existing constructs. For both genes, the constructs replace the entire ORF (2856 bp and 2664 bp for *MSH2* and *MLH1*, respectively) after homologous integration. Cells with a single allele deleted (heterozygous mutants; +/-) were first selected using either a construct encoding resistance to blasticidin (*BSD*) or puromycin (*PUR*) and confirmed by PCR (S2 Fig). The +/- cells were then transformed with the reciprocal construct to attempt to make-/- mutants lacking both alleles; in both cases, and for reasons that are unclear, this was only successful when transforming the *PUR* deletion constructs into the cells that had integrated *BSD* and, furthermore, required a number of transformation attempts using varying antibiotic selection concentrations. Correct integration of the constructs and loss of both *TbMSH2* or *TbMLH1* alleles in the-/- mutants were confirmed by PCR (S2 Fig). Deletion of both alleles in the PCF-/- mutants was further verified by RT-PCR amplification of RNA extracted from drug resistant parasites: as shown in Fig 1A, PCR products from the ORFs were detected in +/- cells as well as in wild type (WT) parasites, but not in the-*/-* mutants.

To generate *T*. *cruzi* epimastigote *msh2*-/- mutants, we started with clones in which one *MSH2* allele was deleted (+/- cells) [37] and replaced by the ORF for the hygromycin phosphotransferase (*HYG*) gene. It should be noted that in the *msh2* heterozygous mutants (+/-) the *HYG* mRNA is generated by the predicted 5’ and 3’ processing signals flanking the *MSH2* gene. In order to mutate the remaining allele, we found it was necessary to use a gene disruption construct (S1 Fig) in which a neomycin phosphotransferase gene ORF (*NEO*) was flanked upstream by processing signals derived from the *T*. *cruzi* intergenic region of the TcP2β gene [52], and downstream by the intergenic region derived from the *gapdh* gene [42]. To allow integration into the remaining *MSH2* allele, homologous targeting was based on *TcMSH2* ORF sequence, and not on the 5’ and 3’ UTRs, as used before to generate the *msh2+*/- mutants. Finally, in order to successfully select for-/- cells, in which the first allele was deleted and the second disrupted, transformed parasites had to be selected initially with a low drug concentration (100 μg.mL^-1^) that was gradually increased to 150 μg.mL^-1^ and 200 μg.mL^-1^ during a period of 4 weeks. PCR amplification of DNA extracted from the *Tcmsh2+/-* cells, and from three cloned putative *Tcmsh2-/-* cell lines showed the expected integration of the different *MSH2* targeting constructs in the *T*. *cruzi* genome (S1 Fig). Northern blots using a probe specific for the *TcMSH2* ORF and for the 24Sα rRNA as a loading control showed 52% less *TcMSH2* mRNA in the *+/-* cells relative to WT, and no detectable *MSH2* mRNA in the *Tcmsh2-/-* clones (Fig 1B), confirming the gene disruption. The apparent reduction in *TcMSH2* mRNA levels in the +/- cells is consistent with the observation of altered phenotypes in these cells relative to WT (see below; and [37]). Similar to *T*. *brucei msh2* mutants, no changes in population doubling times were detected when comparing growth of WT cells to the *T*. *cruzi* mutants with one or both *msh2* alleles deleted (S3 Fig).

**Fig 2:**
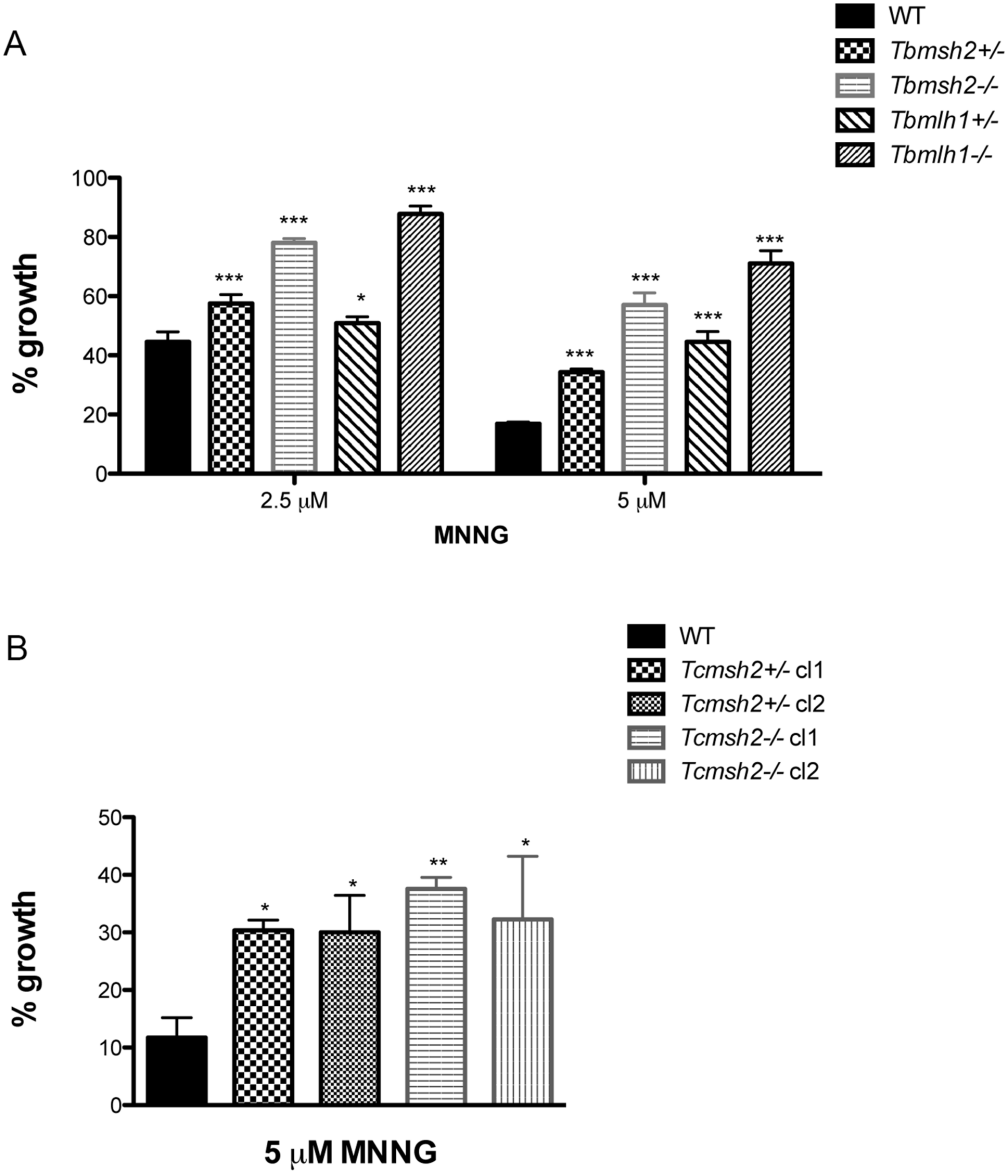
Susceptibility of *T*. *brucei* and *T*. *cruzi* MMR knockout mutants to N-methyl-N’-nitro-N-nitrosoguanidine (MNNG). (**A**) *T*. *brucei* wild type (WT) and procyclic form mutants (*Tbmsh2*+/-, *Tbmsh2*-/-, *Tbmlh1*+/- and *Tbmlh1*-/-) were grown in culture medium with 0 μM, 2.5 μM or 5 μM MNNG. Cell density was measured after 72 hours growth and is plotted as the percentage survival of the MNNG treated cells relative to untreated cultures. (**B**) WT *T*. *cruzi* epimastigotes and MSH2 mutants (*Tcmsh2*+/- and *msh2*-/-) were grown in culture medium with 0 μM or 5 μM MNNG. Cell viability was measured after 72 hours and is plotted as the percentage survival of the MNNG treated cells relative to untreated cultures. Vertical lines indicate standard deviation. ***p<0.001, **p<0.01, *p<0.05: determined by one-way ANOVA with Bonferroni post-test of knockout mutants relative to wild type cells.

To ask if the mutations described above result in detectable loss of MMR, we measured the sensitivity of the parasites to MNNG [26]. *T*. *brucei* and *T*. *cruzi* mutants and WT cells were grown for 72 hours with increasing concentrations of MMNG. Survival of the cells was determined by measuring the cell density of the WT or mutant cells after MNNG treatment relative to untreated cells. In *T*. *brucei*, all cells showed increasing growth impairment as MNNG was increased from 2.5 to 5 μM. However, similar to what has been described in BSF cells [21], and consistent with the proposed futile cycle of alkylation repair in MMR-proficient cells [26, 27], deletion of one allele of *Tbmsh2* or *Tbmlh1* in the PCF+/- mutants caused increased tolerance to MNNG, and this tolerance increased yet further when both alleles were deleted in the-/- mutants (Fig 2A). Deletion of *TbMSH2* or *TbMLH1* in PCF cells also resulted in the-/- parasites displaying increased microsatellite instability, as was observed in *T*. *brucei* BSF mutants, indicating decreased replication fidelity (S4 Fig). In *T*. *cruzi* increased tolerance to 5 μM MNNG was seen in the *Tcmsh2*+/- cells and was not detectably increased in the-/- mutants (Fig 2B). Though this somewhat contrasts with the response of *T*. *brucei* MMR mutants to MNNG, these data nonetheless show that mutation of MMR genes in either parasite resulted in the expected enhanced survival in the presence of this alkylating drug.

**Fig 3:**
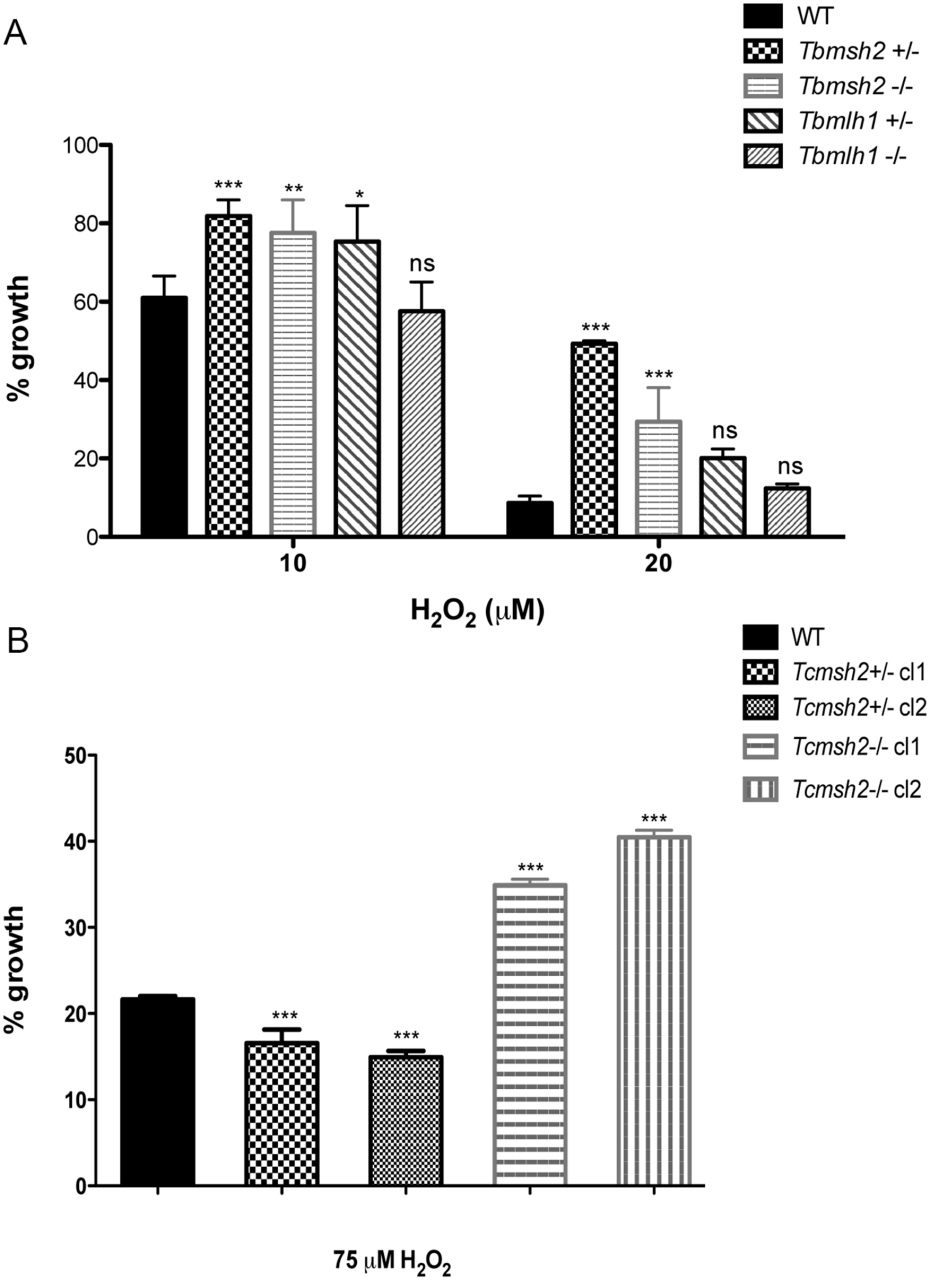
MSH2 knockout mutants are more resistant to oxidative stress generated by H_2_O_2_. (**A**) *T*. *brucei* wild type (WT), *msh2*+/-, *msh2*-/-, *mlh1*+/- and *mlh1*-/- procyclic form cells were grown in culture medium with 0 μM, 10 μM or 20 μM H_2_O_2_. Cell density was measured after 48 hours and plotted as the percentage survival of the H_2_O_2_ treated cells relative to untreated. (**B**) *T*. *cruzi* epimastigote WT, *msh2*+/- and *msh2*-/- cells were incubated with or without 75 μM H_2_O_2_ for 20 minutes in PBS 1x and then allowed to grow in LIT medium for 48 hours, after which cell viability was determined and plotted as percentage survival of the treated cells relative to untreated. Vertical lines show standard deviation. ***p<0.001, **p<0.01, *p<0.05: determined by one-way ANOVA with Bonferroni post-test of mutants relative to wild type cells; ns indicates no signifcant difference.

To ask how the loss of the MMR genes affects the response of *T*. *brucei* and *T*. *cruzi* cells to oxidative stress, we compared growth of the WT cells and mutants in the presence of H_2_O_2_, again determining survival in the presence of the damaging agent relative to undamaged cells. In striking contrast to our previous observations in *T*. *brucei*, which showed that *Tbmsh2*-/- BSF cells are more susceptible to H_2_O_2_ [38], PCF *T*. *brucei msh2* mutants were more resistant to H_2_O_2_ than WT cells after 48 hrs growth at either 10 μM or 20 μM (Fig 3A): improved survival was seen in both the *msh2*+/- and *msh2*-/- cells at this time point, and was also observed 24 and 72 hrs post-treatment (S5 Fig). In contrast, though *T*. *brucei mlh1*+/- mutants displayed a modest increase in H_2_O_2_ resistance (in particular upon treatment with 10 μM H_2_O_2_; Fig 3A), *mlh1*-/- mutants displayed no detectable difference from WT, indicating that the MMR response to H_2_O_2_ damage is predominantly mediated through MSH2 and not MLH1. This separation in function between the two MMR factors is consistent with observations in *T*. *brucei* BSF cells, though with distinct patterns of H_2_O_2_ sensitivity [38]. Analysis of the *T*. *cruzi* epimastigote mutants revealed that loss of both *msh2* alleles also resulted in increased resistance to H_2_O_2_ in these parasite, though loss of one *msh2* allele resulted in increased sensitivity to 75 μM H_2_O_2_, which is distinct from *T*. *brucei msh2*+/- mutants [38] and consistent with previous findings [37] (Fig 3B). Taken together, the enhanced survival of PCF *T*. *brucei msh2* null mutants to H_2_O_2_ exposure suggests a life cycle difference in the consequence of this mutation relative to BSF cells. Moreover, this unexpected phenotype is conserved in epimastigote *T*. *cruzi msh2* null mutants.

### *T*. *cruzi msh2* null mutants have increased survival in macrophages

**Fig 4:**
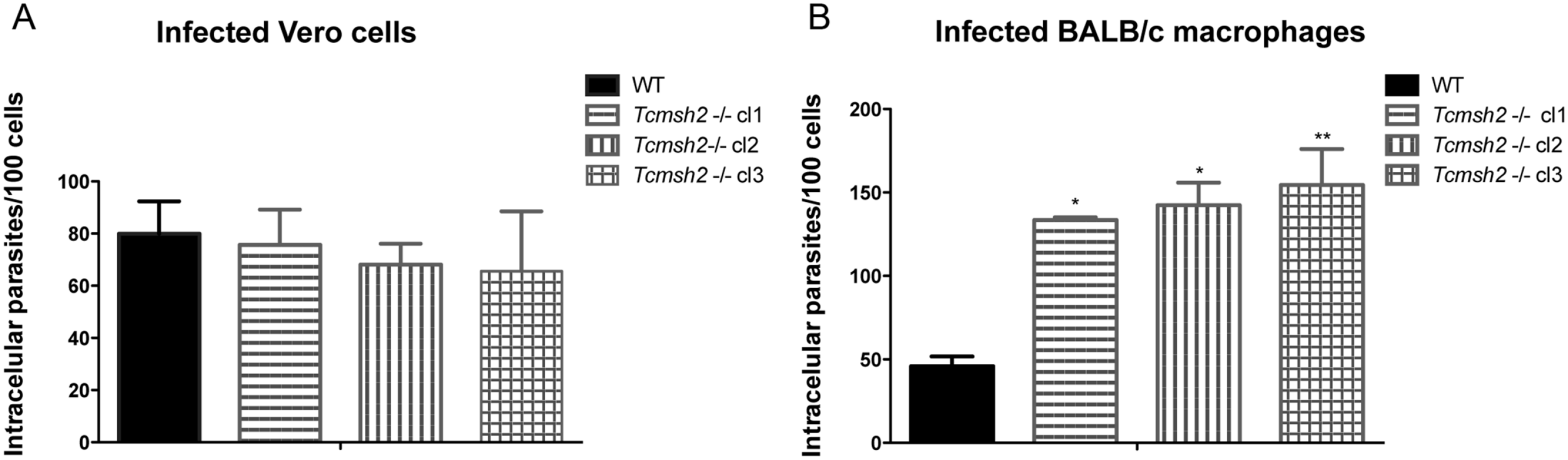
Assessment of *T*. *cruzi msh2* knockout mutant infectivity *in vitro*. (**A**) *T*. *cruzi* trypomastigote cells released by Vero cells infected with either WT or with three cloned cell lines of *Tcmsh2*-/- mutants were counted and equal numbers were used to infect Vero cells attached to glass coverslips or (**B**) cultured intraperitoneal macrophages extracted from BALB/c mice. Graphs show the average number of intracellular parasites counted per 100 cells; vertical lines denote standard deviation. **p<0.01, *p<0.05: one-way ANOVA with Bonferroni post-test of knockout mutants relative to wild type.

To investigate the role of MSH2 in protecting *T*. *cruzi* against oxidative stress generated by host cells, we compared the capabilities of *T*. *cruzi* WT and *msh2-/-* mutants to infect two different cell types: Vero cells and mouse macrophages. Equal numbers of trypomastigotes derived from Vero cells infected with WT and *msh2*-/- mutants were added to cultures of the two cell types and the numbers of intracellular amastigotes were determined 2 days later by staining parasite nuclei with DAPI. As shown in Fig 4A, no difference in the number of intracellular amastigotes was observed in Vero cells infected with WT or *Tcmsh2-/-* parasites. However, infections with each of the three *Tcmsh2*-/- clonal cell lines resulted in a 3-fold increase in the number of intracellular amastigotes relative to infection with WT cells in cultivated mouse macrophages (Fig 4B). As macrophages generate ROS, through an infection-induced respiratory burst [6], while Vero cells do not, these infection data appear consistent with the increased resistance of *T*. *cruzi msh2*-/- null mutants to H_2_O_2_ treatment (see Fig 3B). The infection data also indicate that the increased capacity to survive exposure to oxidative stress is maintained after *T*. *cruzi* differentiates into other life cycle stages.

### *T*. *brucei* and *T*. *cruzi msh2* mutants accumulate 8 oxoguanine in their DNA

**Fig 5:**
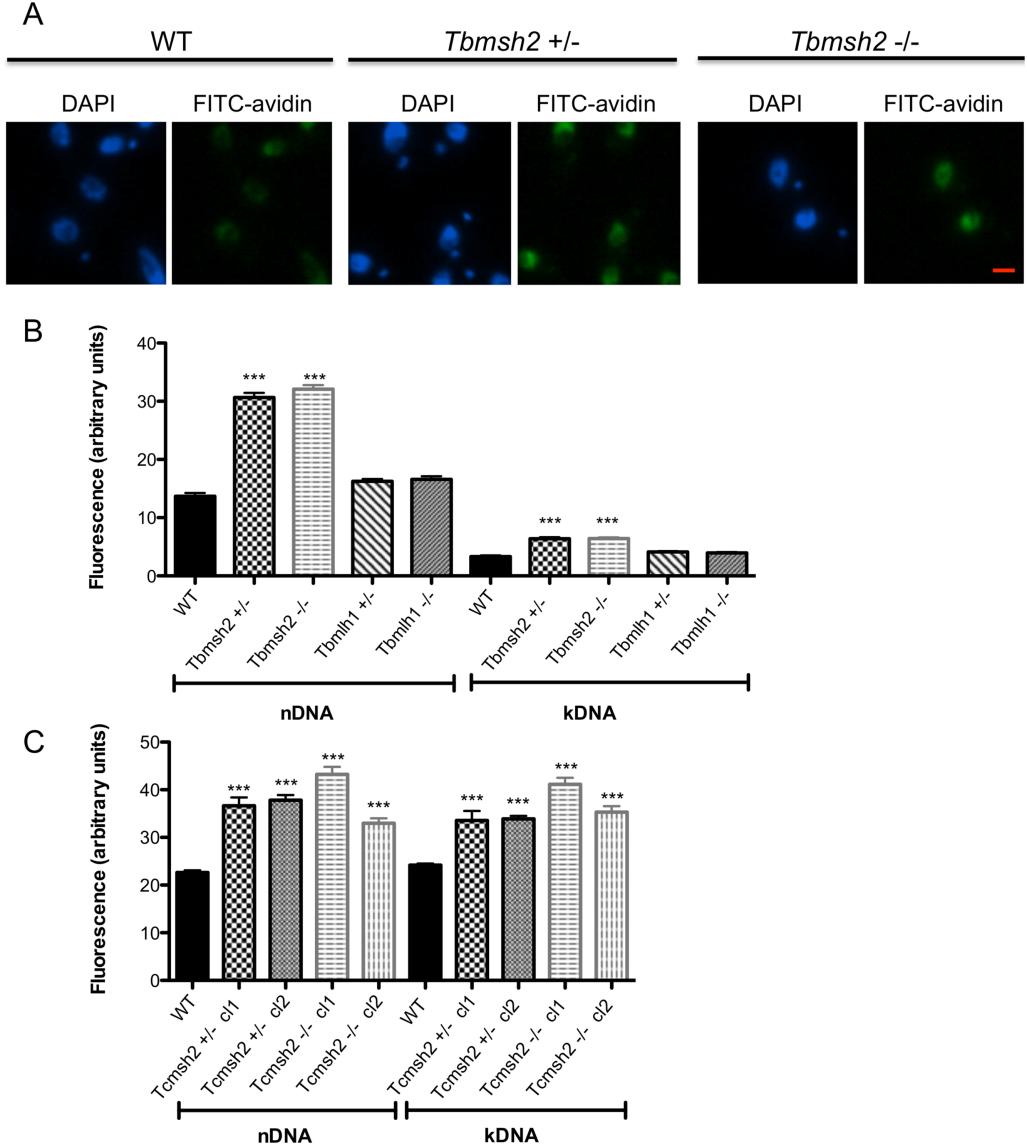
8-oxoguanine (8-oxoG) accumulation in MSH2 knockout cells. FITC-avidin was used to estimate 8-oxoG levels based on the fluorescence intensity in the nuclear DNA (nDNA) and kinetoplast DNA (kDNA) of *T*. *brucei* procyclic forms and *T*. *cruzi* epimastigotes. (**A**) Representative images of FITC-avidin or DAPI stained *T*. *brucei* WT cells and *Tbmsh2*+/- or *Tbmsh2*-/- mutants are shown. (Bar = 1.9 μM) (**B**) Fluorescence intensity of FITC-avidin signals were quantified in the nDNA and kDNA using ImageJ software and plotted as arbitrary units; values shown are the average signal from 100 WT, *Tbmsh2+/-*, *Tbmsh2-/-*, *Tbmlh1+/-* or *Tbmlh1-/-* PCF cells; vertical lines show standard error (SEM). (**C**) FITC-avidin signal evaluated by the same process in *T*. *cruzi* epimastigote WT cells and in *msh2+/-* and *msh2-/-* knockout mutants. ***p<0.001: determined by one-way ANOVA with Bonferroni post-test of knockout mutants relative to wild type; ns indicates no signifcant difference.

The increased resistance to oxidative stress observed in *T*. *brucei* PCF cells and *T*. *cruzi* epimastigotes after MSH2 mutation could be a direct consequence of the loss of this MMR component, or could be due to indirect effects, such as a metabolic adaptation. To begin to address this, we determined the capacity of mutant cells to limit the accumulation of oxidized bases in their genome, which can arise from exposure to ROS generated by endogenous parasite metabolism or from the host. The primary DNA lesion generated by oxidative damage is 8-oxoG, which, if not removed by base-excision repair, can be recognized by MMR [26]. We therefore measured the levels of this oxidized base in the WT and *msh2* mutant parasite genomes using avidin-conjugated FITC and measuring fluorescence [47]. As shown in Fig 5A and 5B, PCF *T*. *brucei msh2*+/- and *msh2*-/- mutants each displayed ~2 fold greater fluorescence in their nuclear DNA (nDNA) and kinetoplast DNA (kDNA) compared with WT cells. These fluorescence data indicate that the levels of 8oxoG were no greater in the *T*. *brucei msh2*-/- mutants than in the *Tbmsh2+/-* mutants, consistent with the observation that the two mutants display equivalent levels of resistance to H_2_O_2_ (Fig 3). In contrast, no difference was observed in the levels of fluorescence in the nDNA or kDNA of *mlh1* mutants related to WT (Fig 5B), a distinction from *T*. *brucei msh2* mutants again consistent with the separation of MMR functions observed when examining levels of resistance to H_2_O_2_. Increased levels of avidin-FITC fluorescence were also seen in the nDNA and kDNA of *T*. *cruzi* epimastigote *msh2* mutants: 1.7 to 1.9 fold increase in fluorescence was seen in the nDNA and kDNA of both *Tcmsh2*+/- clones and both *Tcmsh2-/-* clones examined relative to WT (Fig 5C). These data suggest that loss or reduction of MSH2 expression (but not MLH1) in PCF *T*. *brucei* and in epimastigote *T*. *cruzi* results in the impairment of a pathway involved in the repair of DNA-directed oxidative damage, such as 8-oxoG. Thus, the increased resistance to H_2_O_2_ in the mutants is best explained by an indirect adaptation to cope with oxidative stress following MSH2 mutation.

### Functional complementation confirms adaptation following loss of MSH2 in procyclic form *T*. *brucei* mutants

**Fig 6:**
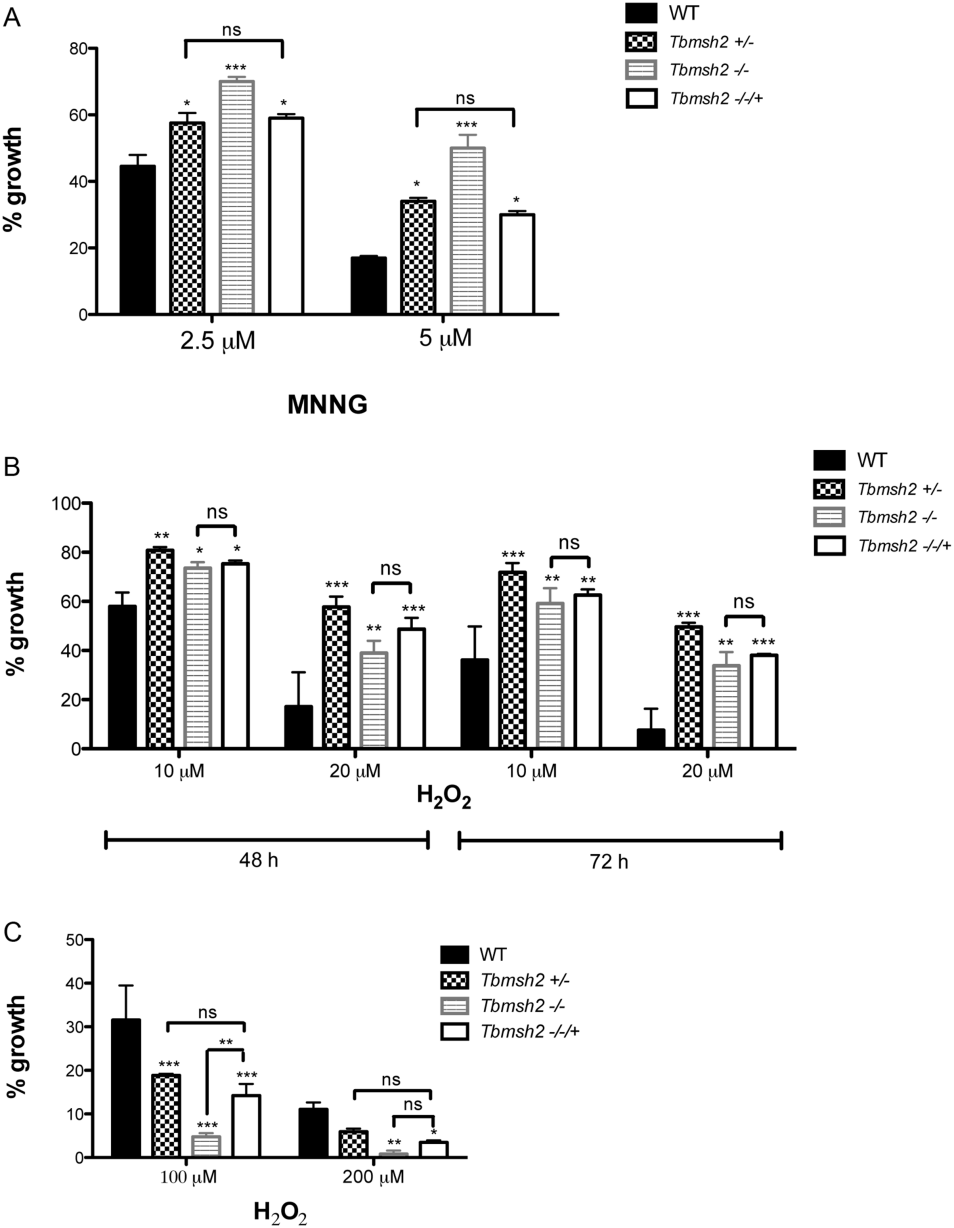
Re-expression of MSH2 in *T*. *brucei msh2* null mutants. (**A**) Procyclic form (PCF) *T*. *brucei* (Tb) wild type (WT), *Tbmsh2*+/-, *Tbmsh2*-/- and *Tbmsh2*-/- cells in which MSH2 is re-expressed (*Tbmsh2*-/-/+) were grown in culture medium with 0 μM, 2.5 μM or 5 μM MNNG. Cell density was measured after 72 hours growth and is plotted as the percentage survival of the MNNG treated cells relative to untreated. (**B**) PCF WT, *Tbmsh2*+/-, *Tbmsh2*-/- and *Tbmsh2*-/-/+ cells were grown in culture medium with 0 μM, 10 μM or 20 μM H_2_O_2_ and cell density was determined 48 and 72 hours later; growth is shown percentage survival of the treated cells relative to untreated (**C**) Growth of wild type bloodstream form (BSF) *T*. *brucei* cells was compared to *Tbmsh2*+/-, *Tbmsh2*-/- and *Tbmsh2*-/-/+ BSF mutants in the presence of 100 μM or 200 μM H_2_O_2_ as described above; graph shows survival of the mutants after 48 hours growth plotting the density of the treated cells as a percentage of the untreated; vertical lines show standard deviation. ***p<0.001, **p<0.001: determined by one-way ANOVA with Bonferroni post-test of mutants relative to wild type; ns indicates no significant difference.

In order to test further if H_2_O_2_ resistance in *msh2* mutants results from an adaptation process that occurred in insect life stages of *T*. *brucei*, we re-expressed MSH2 in both the BSF and PCF *msh2*-/- cells, using a previously described construct and conditions [21]. Integration of the MSH2 re-expression construct was confirmed by PCR (S6 Fig), and southern blot in BSF [21]. Both BSF and PCF *msh2* mutants display increased tolerance to MNNG (Fig 2A), and re-expression of MSH2 in BSF *msh2-/-* mutants reverts this tolerance to the levels of *msh2*+/- mutants [21, 38]. As shown in Fig 6A, re-expressing MSH2 (*Tbmsh2*-/-/+) in the PCF *msh2*-/- mutants also resulted in levels of MNNG survival similar to *msh2*+/- cells. In addition, and as seen in *T*. *brucei* BSF msh2-/-/+ cells [21], PCF msh2-/-/+ cells no longer showed detectable microsatellite variation in clonal growth assays (S7 Fig). Taken together, these assays indicate that MMR function can be restored in *T*. *brucei msh2* null mutants in both life cycle stages after re-introduction of MSH2 into the genome. In contrast, MSH2 re-expression had a different outcome for H_2_O_2_ sensitivity in the two life cycle stages. When *T*. *brucei* PCF *msh2-/-/+* cells were grown in the presence of 10 μM or 20 μM H_2_O_2_ for 48 or 72 hrs, there was no significant difference in survival relative to the *msh2-*/- mutants, and survival was significantly different from the *msh2*+/- mutants (Fig 6B). However, in BSF *T*. *brucei*, the survival of the *msh2*-/-/+ cells (in this case after 48 hrs growth in either 100 or 200 μM H_2_O_2_) was indistinguishable from the *msh2*+/- cells and significantly greater than the *msh2*-/- mutants (Fig 6C). Thus, while re-expression of MSH2 in *T*. *brucei msh2*-/- null mutants was able to restore MMR function in both life cycle stages, the same re-expression was able to revert the increased sensitivity to H_2_O_2_ only in BSF *msh2*-/- mutants. In contrast, the increased tolerance to H_2_O_2_ observed in PCF after loss of MSH2 could not be reverted by re-expressing this gene in this life cycle form of *T*. *brucei*. This lack of MSH2 complementation is most simply explained by changes in expression or function of another factor(s) that allowed PCF MSH2 mutants to cope specifically with oxidative stress, though we cannot rule out the possibility of this specific phenotypic difference between BSF and PCF cells arising due to differing levels of MSH2 in the two life cycle stages and in the *msh2*-/-/+ re-expressers. As suggested by RNAi data (S8 Fig), the adaptation process that may have occurred during the cloning period needed to generate the *msh2*-/- mutants requires several generations. When MSH2 expression is abruptly inhibited by tetracycline induction of siRNA in *T*. *brucei* PCF cells, a discernible slowing of growth is observed as a consequence of the reduced *msh2* mRNA expression.

### *T*. *brucei* and *T*. *cruzi* **MSH2 display nuclear localization**

**Fig 7:**
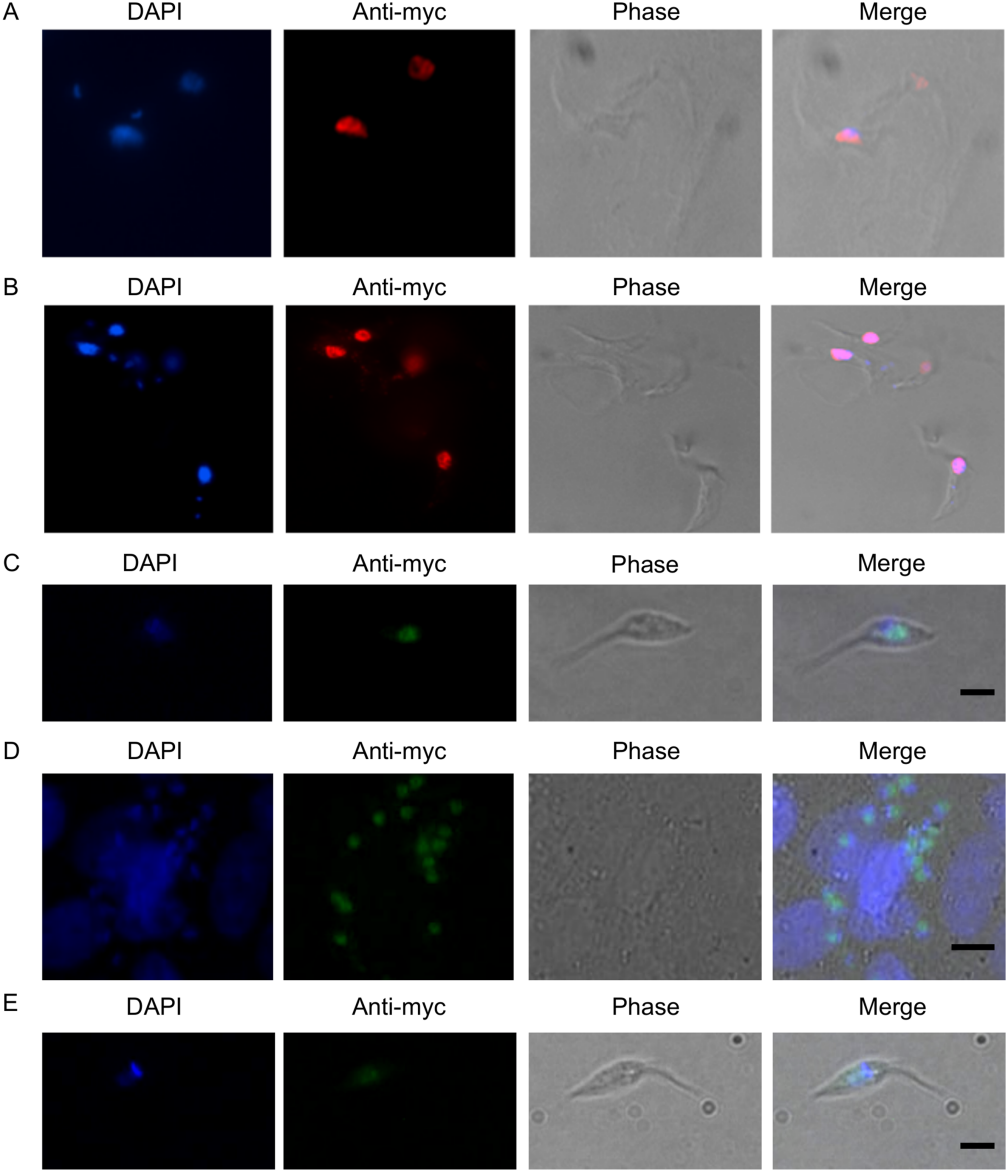
MSH2 displays nuclear localisation in *T*. *brucei* and *T*. *cruzi*. Parasites expressing MSH2 C-terminally fused to 12 copies of the MYC epitope were used for immunolocalization of MSH2 using anti-MYC antiserum. (**A**) Transfected *T*. *brucei* bloodstream forms, (**B**) *T*. *brucei* procyclic forms, (**C**) *T*. *cruzi* epimastigotes, (**D**) *T*. *cruzi* intracellular amastigote cells inside infected Vero cels and (**E**) *T*. *cruzi* epimastigotes after 20 min treatment with 70μM H_2_O_2_ are shown. Representive images of DAPI staining of DNA, immunoflourescence with anti-MYC antiserum and phase contrast images of parasite cells are shown together with merged images. (Bars = 2.3 μm).

In this study, and in previous work [37], we have shown that deletion of *msh2* results in increased levels of 8-oxoG in both the nDNA and kDNA. Indeed, in BSF *T*. *brucei* the most pronounced cell cycle effect of MSH2 loss, which is exacerbated by H_2_O_2_ treatment, is the accumulation of cells with reduced amounts of kDNA (evaluated by DAPI staining) [37]. Because of these findings, and coupled with lack of a detectable kinetoplastid homologue of MSH1 (a MutS-like factor involved in mitochondrial genome repair, including oxidative damage repair) [53, 54], we hypothesized that MSH2 could provide a repair function for the trypanosomes’ mitochondrial (kDNA) genome. To test this hypothesis, we expressed *T*. *brucei* and *T*. *cruzi* MSH2 as C-terminal fusions with a 12X c-MYC epitope. For *T*. *brucei*, MSH2-myc was expressed from the endogenous locus in both BSF and PCF cells in which the other *MSH2* allele was deleted; by evaluating MNNG sensitivity, we showed that the epitope tag did not affect the function of MSH2 in BSF cells (S9 Fig). As shown Fig 7A and 7B, immunolocalization of MSH2:myc with anti-c-MYC antiserum in *T*. *brucei* BSF and PCF cells revealed only a nuclear signal. The same nuclear localization was found in *T*. *cruzi* epimastigotes expressing MSH2:myc, as well as in amastigotes obtained after infection of Vero cells (Fig 7C and 7D). No changes in subcellular localization of the tagged MSH2 protein were observed in *T*. *cruzi* epimastigotes expressing MSH2:myc after exposure to H_2_O_2_ (Fig 7E) or other genotoxic agents, such as MNNG and cisplatin.

## Discussion

In previous studies we have described the phenotypic effects of deleting both alleles of *MSH2* or *MLH1* in *T*. *brucei* BSF cells, and the effects of loss of a single allele of *MSH2* in *T*. *cruzi* epimastigotes [21, 37]. Here, we provide several new insights into the function of MMR in African and American trypanosomes. First, we show that genetic ablation of MSH2 or MLH1 is possible in *T*. *brucei* PCF cells, as is MSH2 ablation in *T*. *cruzi* epimastigotes. In all cases, loss of MSH2 results in detectable impairment in MMR, as demonstrated by the increased tolerance to the alkylator MNNG, a phenotype specifically seen in MMR mutants in other eukaryotes [55] and in *T*. *brucei* BSF cells [21]. Second, we reveal a striking life cycle dependent difference in the effect of MSH2 ablation in *T*. *brucei*: in PCF cells loss of MSH2 results in increased tolerance to H_2_O_2_, whereas MSH2 loss results in increased sensitivity in BSF cells [38]. The same increased tolerance is also seen in *T*. *cruzi* epimastigote MSH2 mutants, where it impacts on the capacity of the parasite to grow in ROS-producing host macrophages. Importantly, altered H_2_O_2_ resistance was not observed when *T*. *brucei* BSF or PCF cells lacking MLH1 were examined. Finally, we provide evidence that the increased resistance to H_2_O_2_ in *T*. *brucei* after loss of MSH2 is due to a life cycle dependent adaptation in a facet of the cell that is distinct from MSH2, since re-expression of the protein restores MMR activity in PCF cells but does not alter the response to H_2_O_2_. In contrast, re-expression of MSH2 in *T*. *brucei* BSF *msh2*-/- mutants reverts both MMR impairment and H_2_O_2_ sensitivity.

Despite the complexity of phenotypes observed after MMR mutation, the findings indicate a common function of MSH2 in both trypanosomes: in addition to its role as a key component of MMR, MSH2 is also directly involved in the response to oxidative stress. Since loss of MLH1 did not result in increased resistance to H_2_O_2_ in PCF *T*. *brucei*, our new data confirms previous indications from *BSF T*. *brucei* [38] that although MSH2 acts in the parasite’s response to oxidative stress, such a role is unlikely to involve execution of MMR on oxidative lesions through MLH1-directed functions. Indeed, this is supported by the observation that PCF *T*. *brucei* MSH2 mutants, but not MLH1 mutants, display increased levels of 8-oxoG, a known form of oxidized base damage [56], in their nuclear and mitochondrial genomes. The same accumulation of 8-oxoG is also seen in *T*. *cruzi msh2*-/- and *msh2*+/- mutants [37], indicating that such a role for MSH2 is conserved in the two trypanosomes species. Most likely, a separation of function between MSH2 and MLH1 in this role will also be found in *T*. *cruzi*, though this has not to date been tested. The nature of MSH2’s role in responding the oxidative damage remains unclear, including whether or not it is limited to acting upon or repairing 8-oxoG. One possibility is that MSH2 acts in conjunction with MSH6 to repair 8-oxoG in DNA, a known activity of this heterodimer in other eukaryotes that can act independently of downstream MMR components [32]. However, we also observe accumulation of 8-oxoG in the kDNA of *T*. *brucei* and *T*. *cruzi* MSH2 mutants, consistent with previous descriptions of kDNA loss in *T*. *brucei* BSF *msh2*-/- mutants [37], and it is not clear that MSH2-6 could direct such a repair role in the mitochondrion. A broader role for MSH2 in the oxidative stress response might therefore need to be considered, perhaps related to findings in other eukaryotes that MSH2-6 can act to signal the presence of various forms of DNA damage, for instance through ATR [57–59]. It remains puzzling, however, that much of the signaling and wider activities attributed to MSH2-6 in other eukaryotes appear to reside in the extended N-terminal domain of MSH6 [59], which is notably truncated in trypanosomatid MSH6 orthologues [21]. Nonetheless, such a role for MSH2 perhaps most readily explains the adaptation we propose in *T*. *brucei* PCF, but not in BSF cells after loss of MSH2. PCF *T*. *brucei* have active mitochondrial metabolism, unlike BSF, and therefore greater levels of endogenous ROS, meaning that loss of MSH2 may be more detrimental in this life cycle stage [39]. *T*. *cruzi* epimastigotes also have an aerobic metabolism relying on mitochondrial respiration [60], and it is thus tempting to speculate that the same adaptation most likely occurred during the generation of *msh2* null mutants in *T*. *cruzi* epimastigotes; though we have no direct evidence for such adaptation, the selection of cells lacking both alleles was notably problematic and necessitated an altered targeting transfection approach for removing the second allele.

To date, our analysis of the consequences of *T*. *brucei* MMR mutation have only been conducted *in vitro*, meaning it remains possible that the life cycle differences, and in particular the proposed PCF adaptation to MSH2 loss, are due to culture conditions rather than environmental and metabolic changes as the parasite cycles between host and vector. However, we provide direct evidence that the increased capacity to survive oxidative stress observed in *T*. *cruzi msh2* -/- mutants may reflect challenges faced by this parasite during its life cycle. Although no difference in infectivity was observed between *T*. *cruzi* WT cells and *msh2-/-* mutants in Vero cells, macrophages infected with *T*. *cruzi msh2-/-* mutants contained almost 3-fold more intracellular amastigotes than macrophages infected with WT parasites. Due to their phagocytic nature, macrophages naturally produce ROS through the activation of NADPH oxidase during phagocytosis or by stimulation with a wide range of infective agents [61]. Although ROS are expected to be involved in pathogen elimination, increasing evidence suggests that ROS production actually could enhance *T*. *cruzi* infection in macrophages. For instance, it was recently proposed that oxidative stress mobilizes cellular iron and fuels *T*. *cruzi* infection [62, 63]. A role for ROS as a signalling molecule has also been highlighted in studies with *Leishmania*, where low ROS concentrations regulate proliferation and differentiation by modulating the activity of cellular targets by oxidation [64]. It seems reasonable to assume that MSH2 is part of the arsenal of functions that trypanosomatid parasites are equipped with to withstand and detect oxidative stress during their natural life cycles, and the absence of MSH2 can have wider effects than simply loss of MMR, as reflected in the adaptation we suggest in PCF *T*. *brucei*.

What is the nature of the adaptation following MSH2 mutation? Answering this question is complicated by the multiple potential pathways that might be affected. One possibility is that loss of MSH2 necessitates the up-regulation of an alternative repair pathway, such as base excision repair (BER). BER may be an attractive option because it is known to respond to oxidative stress and, indeed, a DNA glycosylase termed OGG1 is found in many organisms [65–67], including *T*. *cruzi* [68], that acts on 8-oxoG. However, increased BER activity seems incompatible with the increased levels of 8 oxoG we detect in *T*. *brucei* and *T*. *cruzi* MSH2 mutants. These data indicate that increased oxidative damage to nucleic acids occurs in the mutants and therefore suggests that the cells might adapt by limiting their exposure to oxidative stress in other ways. Trypanosomes have a complex set of enzymes involved in the oxidative stress response. One such pathway distinct from the mammalian host is based on trypanothione metabolism [69]. A paradox in our study is that we can only find evidence for a nuclear localization of MSH2, even after exposure to H_2_O_2_. Most oxidative damage is likely to occur in the trypanosome mitochondrion, as this is the major source of ROS. However, if MSH2 is limited to the nucleus, then how does loss of MSH2 result in (as noted above) increased levels of 8 oxoG in the kDNA? Moreover, if even some of the adaptation we see after loss of MSH2 affects trypanothione metabolism, then how is the loss of a nuclear protein communicated to these enzymes, which are thought to be mitochondrial or cytoplasmic [70–74]? One possibility is that C-terminal tagging impairs mitochondrial localization or MSH2 function. In BSF cells, we can rule this out because MSH2-myc was shown to be functional in the response to MNNG treatment (S9 Fig). However, the same analysis has not been conducted in PCF *T*. *brucei*, where perhaps mitochondrial localization is more important or pronounced. An alternative explanation is that trypanosome MSH2, perhaps in conjunction with MSH6, does indeed provide a role in signaling damage, similar to the activities detailed in other eukaryotes. Whether such signaling is limited to oxidative damage is unknown, but the signal provided by MSH2 may be communicated, in ways still to be determined, beyond the nucleus. Hence, if MSH2 is ablated, this element of the signaling cascade is lost. In the insect stages of at least *T*. *brucei* this loss is more deleterious than in the mammal, necessitating the selection for metabolic adaptation to deal with an altered oxidative stress response, with the consequence that the mutant becomes more resistant to ROS. The same hypothesis applies to *T*. *cruzi* since similar phenotypic changes were also seen in *T*. *cruzi* epimastigote *MSH2* null mutants.

Clearly, further studies will be needed to understand how the parasites adapt to MSH2 mutation and it may be that metabolomic or proteomic approaches are the best strategies to take, in particular if no compensatory genetic changes follow *MSH2* mutation. Nonetheless, this work reveals surprising complexity in the outcome of mutation to a core DNA repair pathway, suggesting interconnections with other cellular pathways. In addition, this study potentially reveals life cycle-dependent differences in MMR function. Loss of BRCA2 has also been shown to have different effects on genome stability in *T*. *brucei* BSF and PCF cells [75], but the extent to which the wide range of DNA repair pathways in trypanosomatids can vary in their activity or use through the life cycle remains little explored.

## Supporting Information

S1 Fig Strategy to knockout *MSH2* or *MLH1* in *T*. *brucei* and *T*. *cruzi*. (**A**) *T*.*brucei (Tb) MSH2* or *MLH1* genes were deleted by replacement of the coding sequence (CDS) of the gene with a DNA construct that consisted of Puromycin (PUR^R^) or Blasticidin (BSD^R^) resistance genes flanked by processing sequences (providing signals for splicing and poliadenylation) derived from the αβ tubulin intergenic regions. Homologous integration was guided by 5’ and 3’ UTR sequences of either gene that surrounded the antibiotic resistance cassettes. The two alleles of *T*.*cruzi MSH2* were each deleted by homologous recombination using different DNA constructs. The first allele was deleted by using 5’ and 3’ UTR sequences to guide integration of the CDS for the Hygromycin resistance gene (Hygro^R^); in this case, splicing and polyadenylation of the resistance gene is assumed to be derived from endogenous MSH2 processing signals. For the second allele, the CDS was not deleted in its entirety, but instead it is was partly deleted by a construction containing a Neomycin resistance gene (Neo^R^) flanked by splicing and polyadenylation signal sequences derived from *HX1* and *GAPDH* intergenic regions containing signals for splicing and poliadenylation. Integration of the resistance cassette here is guided by 5’ and 3’ *MSH2* CDS sequence. (**B**) A PCR screen to identify double knockout clones (-/-) in which both alleles of *TcMSH2* have been targeted was conducted with specific primers annealing in the Hygro^R^ or Neo^R^ resistance genes together with common primers that recognised sequences that flanked the MSH2 fragment included in the the constructs. An agarose gel is shown of the PCR products generated using genomic DNA from *T*. *cruzi* wild type cells (WT), first allele mutant clones (cl1 and cl2 +/-) in which Hygro^R^ has been integrated, or double allele mutants (cl1 and cl2 -/-) in which both Hygro^R^ and Neo^R^ have integrated. c- indicates a control reaction without any input genomic DNA.
(TIF)

S2 Fig Assessing the integration of DNA constructions to delete both TbMSH2 and TbMLH1 alleles. PCR amplifications were carried out to verify the knockout of both *TbMSH2* **(A)** or *TbMLH1* alleles **(B)** using specific primers annealing in the target gene or the PUR^R^ or BSD^R^ resistance genes together with common primers that recognised sequences that flanked the MSH2 or MLH1 fragment included in the the constructs. Arrows shown in the above panels denote regions of the wild type or mutated loci complementary to the different primers. PCR products generated using genomic DNA from *T*. *brucei* wild type cells (WT), PUR mutant clones (*Tbmsh2*/*Tbmlh1* +/-) in which PUR^R^ has been integrated, BSD mutant clones (*Tbmsh2*/*Tbmlh1* +/-) in which BSD^R^ has been integrated or double allele mutants (*Tbmsh2*/*Tbmlh1*-/-) in which both PUR^R^ and BSD^R^ have integrated are shown after separation on agarose gels.
(TIF)

S3 Fig Wild type *T*. *cruzi* epimastigotes and MSH2 knockout mutants have no difference in cell growth *in vitro*. WT *T*. *cruzi* epimastigote and two clonal cell lines with one (*Tcmsh2*+/-) or both *msh2* alleles disrupted (*Tcmsh2*-/-) were diluted in LIT medium to 1x10^7^ parasites.mL^-1^ and grown for additional 3 days. Cell densities were measured at 24 hours intervals; bars indicate standard deviation.
(TIF)

S4 Fig Microsatellite instability in *T*. *bruce*i MMR knockouts. PCR amplification of the microsatellite locus JS2 from PCF WT, *Tbmsh2+/-*, *Tbmsh2-/-*, *Tbmlh1+/-*, and *Tbmlh1-/-* grown in the absence (-) and presence (+) of 20μM H_2_O_2_ for 48 hours and then cloned by limiting dilution in 96 well culture dishes. The JS2 locus was amplified from 10 clones from each cell line using primers JS2A and JS2B [21]. PCR products were separated on 3% agarose gesl; note, the two alleles of this JS2 locus have distinct sizes. PCR product from one of the wild type sub clones was also added in the first and last lane parallel to the mutants, for size comparison. Clones that show a difference in size relative to WT are indicated by an arrow; size markers are shown.
(TIF)

S5 Fig MSH2 knockout mutants in *T*. *brucei* are more resistant to oxidative stress generated by H_2_O_2_ at different time points. *T*. *brucei* wild type (WT), *msh2*+/-, *msh2*-/-, *mlh1*+/- and *mlh1*-/- procyclic form cells were grown in culture medium with 0 μM, 10 μM or 20 μM H_2_O_2_. Cell density was measured after 24 and 72 hours and plotted as the percentage survival of the H_2_O_2_ treated cells relative to untreated; vertical lines show standard deviation. ***p<0.001, **p<0.001, difference between mutants relative to wild type.
(TIF)

S6 Fig Reintegration of *MSH2* in *T*. *brucei* procyclic form (PCF) *msh2-/-* mutants. The upper diagram shows that the gene knockout strategy deletes the entire Tb*MSH2* ORF and indicates the position of primers (‘ORF’) used to test for the presence or absence of *MSH2* ORF in genomic DNA of wild type (WT) cells, *Tbmsh2*+/- and *Tbmsh2*-/- mutants, and in three PCF *Tbmsh2-/-/+* clones (1–3) in which the *MSH2* ORF was reintegrated into the endogenous locus of the-/- mutant using a construct described previously [21]. The lower diagram shows two agarose gels, the uppermost displaying PCR products generated using the ORF primers and the different cells, relative to a control PCR (lower gel) in which the unaltered *MSH2* 5’ UTR was PCR-amplified (‘UTR’); size markers are shown (kb).
(TIF)

S7 Fig Analysis of JS2 microsatellite instability in *T*. *brucei* PCF cells in the presence or absence of of 20 μM H_2_O_2_. WT, *Tbmsh2+/-*, *Tbmsh2-/-* and *Tbmsh2-/-/+* cells were grown in the absence (-) or presence (+) of 20 μM H_2_O_2_ for 48 hours and then cloned by limiting dilution in 96 well culture dishes. The JS2 locus was amplified from 10 clones from each cell line using primers JS2A and JS2B [21] and PCR products were separated on 3% agarose gel; note, the two alleles of the JS2 locus are distinct sizes and are marked by a dashed red line (‘c’ indicates a control PCR reaction run without genomic DNA). PCR product from one of the untreated wild type sub clones (wt-) was added in the first and last lane of the gels showing the mutants, for size comparison. Clones that show a difference in size relative to WT are indicated by an arrow; size markers are shown (bp).
(TIF)

S8 Fig RNAi of MSH2 in *T*. *brucei* PCF. **(A)** Growth curve of *T*. *brucei* PCF cells in which RNAi against MSH2 was induced by the presence of tetracycline (Tet+) (2 μg.ml^-1^) Cell density was measured every 24 hours over a period of 216 hours and compared between the Tet+ cells and PCF cells grown in the absence of tetracycline (Tet-).; the cultures were diluted to their starting density every 72 hours. The graph shows the average cell density of two independent clones, repeated twice; vertical lines indicate standard error. P<0.001 **(B)** mRNA levels of MSH2 and MLH1 were determined in one cloned *T*. *brucei* PCF cell line before and after RNAi induction by quantitative reverse-transcriptase PCR. Total RNA extracted from RNAi-induced (Tet+) and non-induced (Tet-) *T*. *brucei* PCF cells was reverse transcribed and quantified using GPI8 mRNA as an endogenous control. Error bars are standard deviations for four replicates. ***P<0.001
(TIF)

S9 Fig Parasites expressing MSH2:c-myc have a functional protein. *T*. *brucei* BSF *Tbmsh2*+/- were transfected to express MSH2 C-terminally fused to a c-myc tag (*Tbmsh2*:myc) and the MMR efficiency was compared in these cells to *T*. *brucei* wild type (WT), *Tbmsh2*+/- and *Tbmsh2*-/- cells after treatment with MNNG. Cells were grown at a starting density of 1 x 10^5^ cells.ml^-1^ in the presence of increasing concentrations of MNNG (from 0.39 μM—400 μM) in fluorescence-readable 96 well plates. After 48 hours, Alamar Blue was added to each well and fluorescence was measured after a further 24 hours of growth. IC50 values were determined and are shown as the mean of three experiments, with standard deviations indicated by vertical bars.
(TIF)
