# Supplementary figures and images for: Distinct Phenotypes Caused by Mutation of MSH2 in Trypanosome Insect and Mammalian Life Cycle Forms Are Associated with Parasite Adaptation to Oxidative Stress
Source: PLoS Negl Trop Dis. 2015 Jun 17;9(6):e0003870. doi: 10.1371/journal.pntd.0003870 (PMC4470938; doi:10.1371/journal.pntd.0003870)

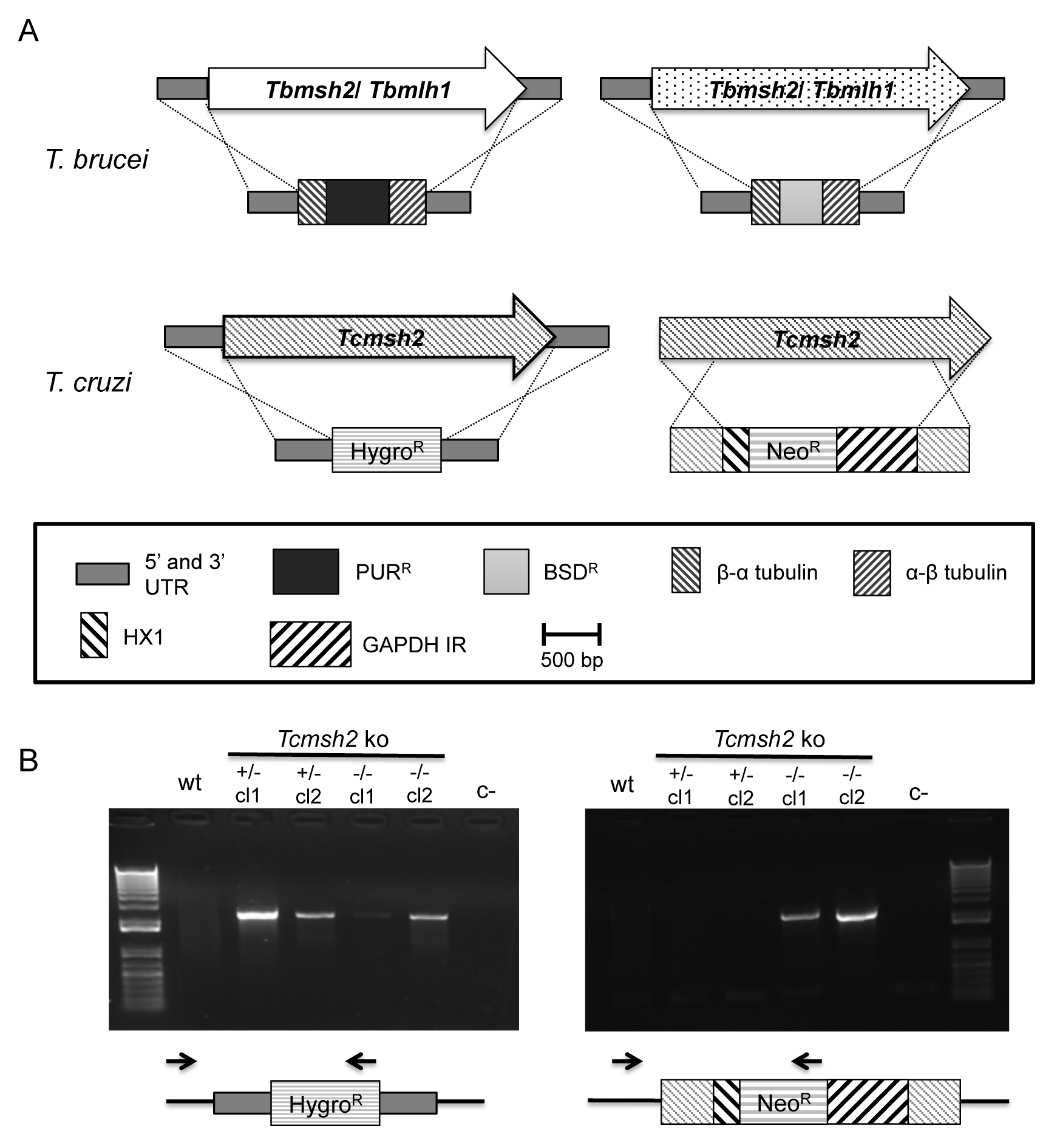

Supplement: S1 Fig — (A) T.brucei (Tb) MSH2 or MLH1 genes were deleted by replacement of the coding sequence (CDS) of the gene with a DNA construct that consisted of Puromycin (PURR) or Blasticidin (BSDR) resistance genes flanked by processing sequences (providing signals for splicing and poliadenylation) derived from the αβ tubulin intergenic regions. Homologous integration was guided by 5’ and 3’ UTR sequences of either gene that surrounded the antibiotic resistance cassettes. The two alleles of T.cruzi MSH2 were each deleted by homologous recombination using different DNA constructs. The first allele was deleted by using 5’ and 3’ UTR sequences to guide integration of the CDS for the Hygromycin resistance gene (HygroR); in this case, splicing and polyadenylation of the resistance gene is assumed to be derived from endogenous MSH2 processing signals. For the second allele, the CDS was not deleted in its entirety, but instead it is was partly deleted by a construction containing a Neomycin resistance gene (NeoR) flanked by splicing and polyadenylation signal sequences derived from HX1 and GAPDH intergenic regions containing signals for splicing and poliadenylation. Integration of the resistance cassette here is guided by 5’ and 3’ MSH2 CDS sequence. (B) A PCR screen to identify double knockout clones (-/-) in which both alleles of TcMSH2 have been targeted was conducted with specific primers annealing in the HygroR or NeoR resistance genes together with common primers that recognised sequences that flanked the MSH2 fragment included in the the constructs. An agarose gel is shown of the PCR products generated using genomic DNA from T. cruzi wild type cells (WT), first allele mutant clones (cl1 and cl2 +/-) in which HygroR has been integrated, or double allele mutants (cl1 and cl2 -/-) in which both HygroR and NeoR have integrated. c- indicates a control reaction without any input genomic DNA. (TIF) [file pntd.0003870.s001.tif]

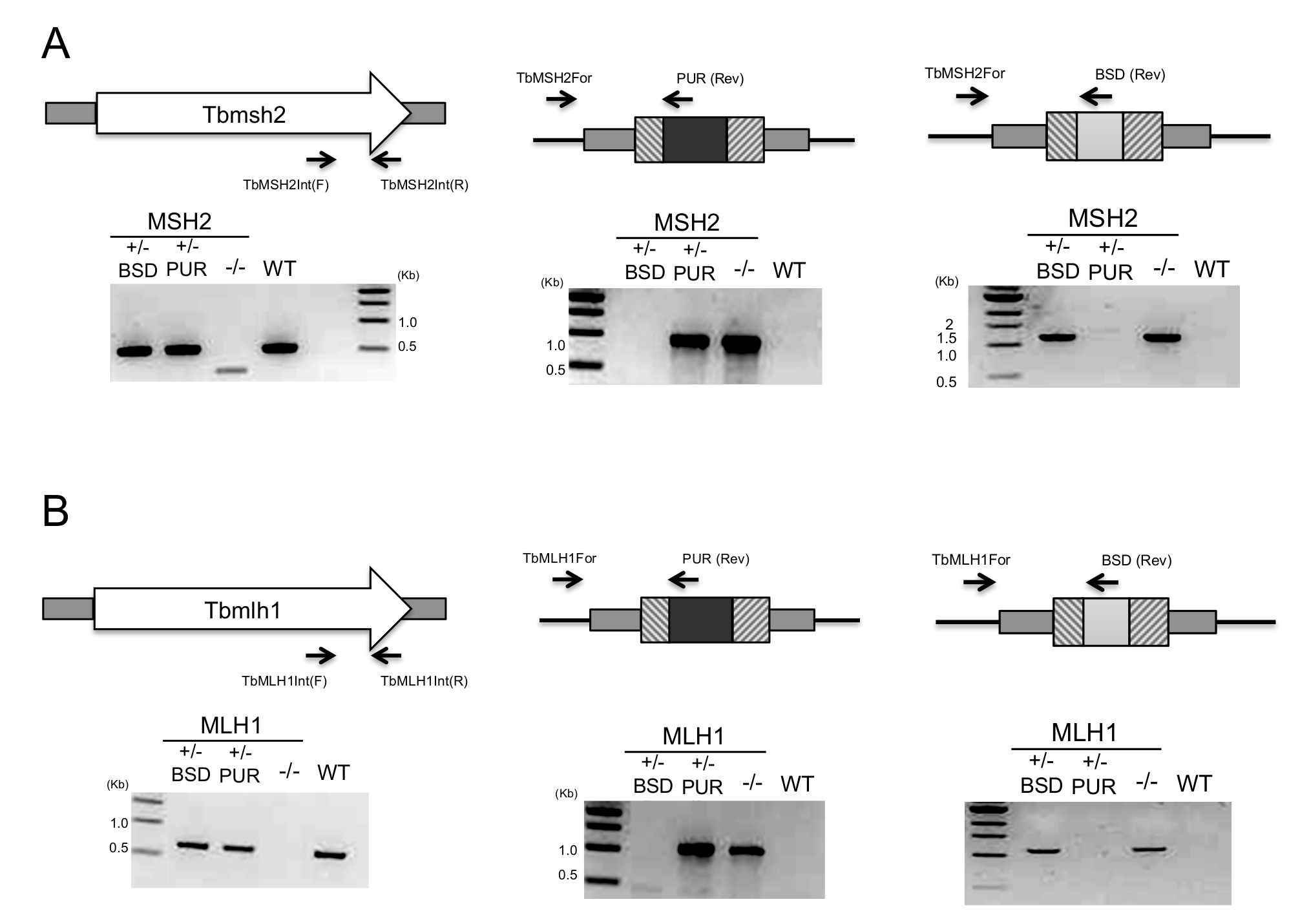

Supplement: S2 Fig — PCR amplifications were carried out to verify the knockout of both TbMSH2 (A) or TbMLH1 alleles (B) using specific primers annealing in the target gene or the PURR or BSDR resistance genes together with common primers that recognised sequences that flanked the MSH2 or MLH1 fragment included in the the constructs. Arrows shown in the above panels denote regions of the wild type or mutated loci complementary to the different primers. PCR products generated using genomic DNA from T. brucei wild type cells (WT), PUR mutant clones (Tbmsh2/Tbmlh1 +/-) in which PURR has been integrated, BSD mutant clones (Tbmsh2/Tbmlh1 +/-) in which BSDR has been integrated or double allele mutants (Tbmsh2/Tbmlh1-/-) in which both PURR and BSDR have integrated are shown after separation on agarose gels. (TIF) [file pntd.0003870.s002.tif]

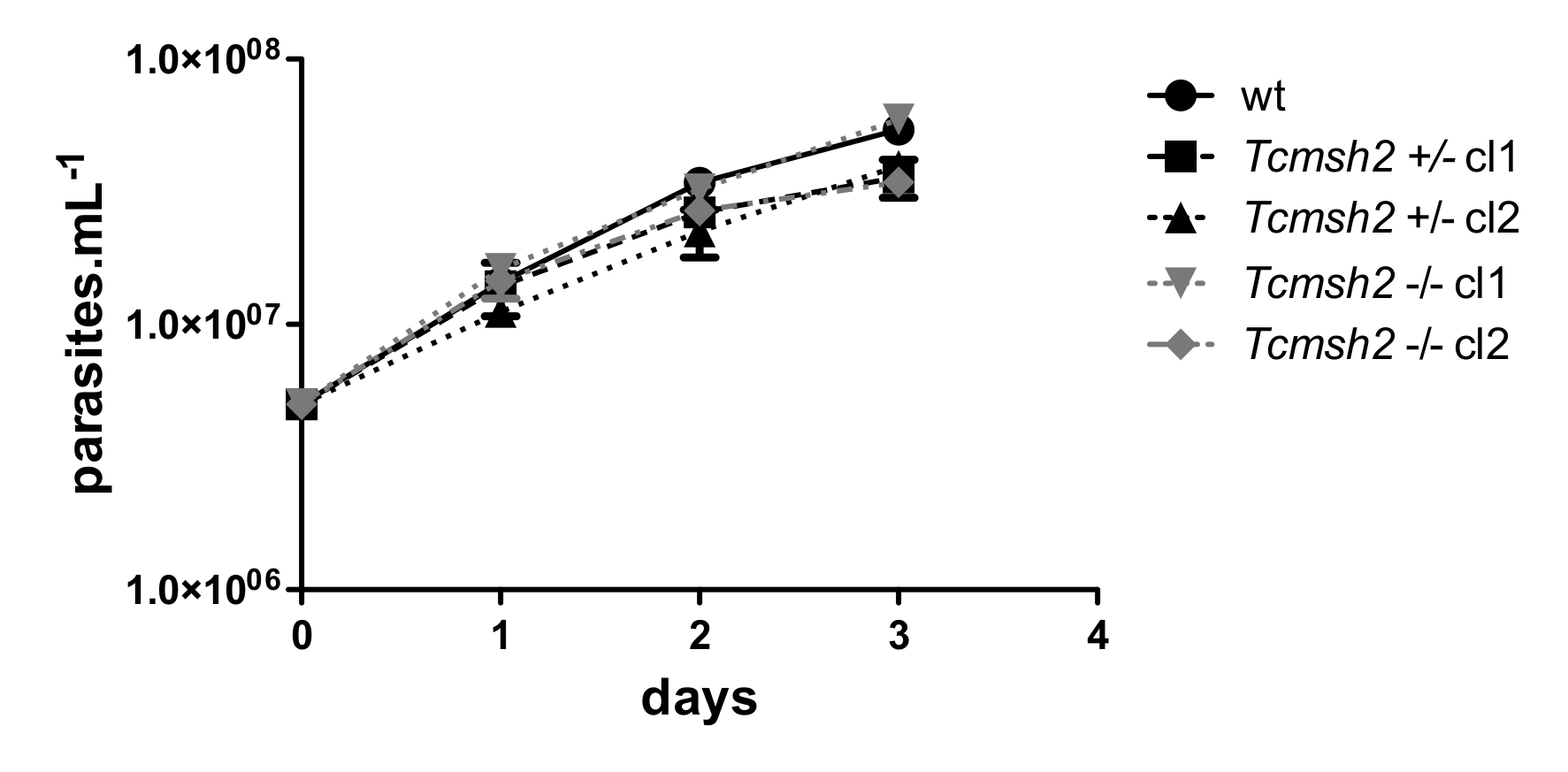

Supplement: S3 Fig — WT T. cruzi epimastigote and two clonal cell lines with one (Tcmsh2+/-) or both msh2 alleles disrupted (Tcmsh2-/-) were diluted in LIT medium to 1x10^7^ parasites.mL-1 and grown for additional 3 days. Cell densities were measured at 24 hours intervals; bars indicate standard deviation. (TIF) [file pntd.0003870.s003.tif]

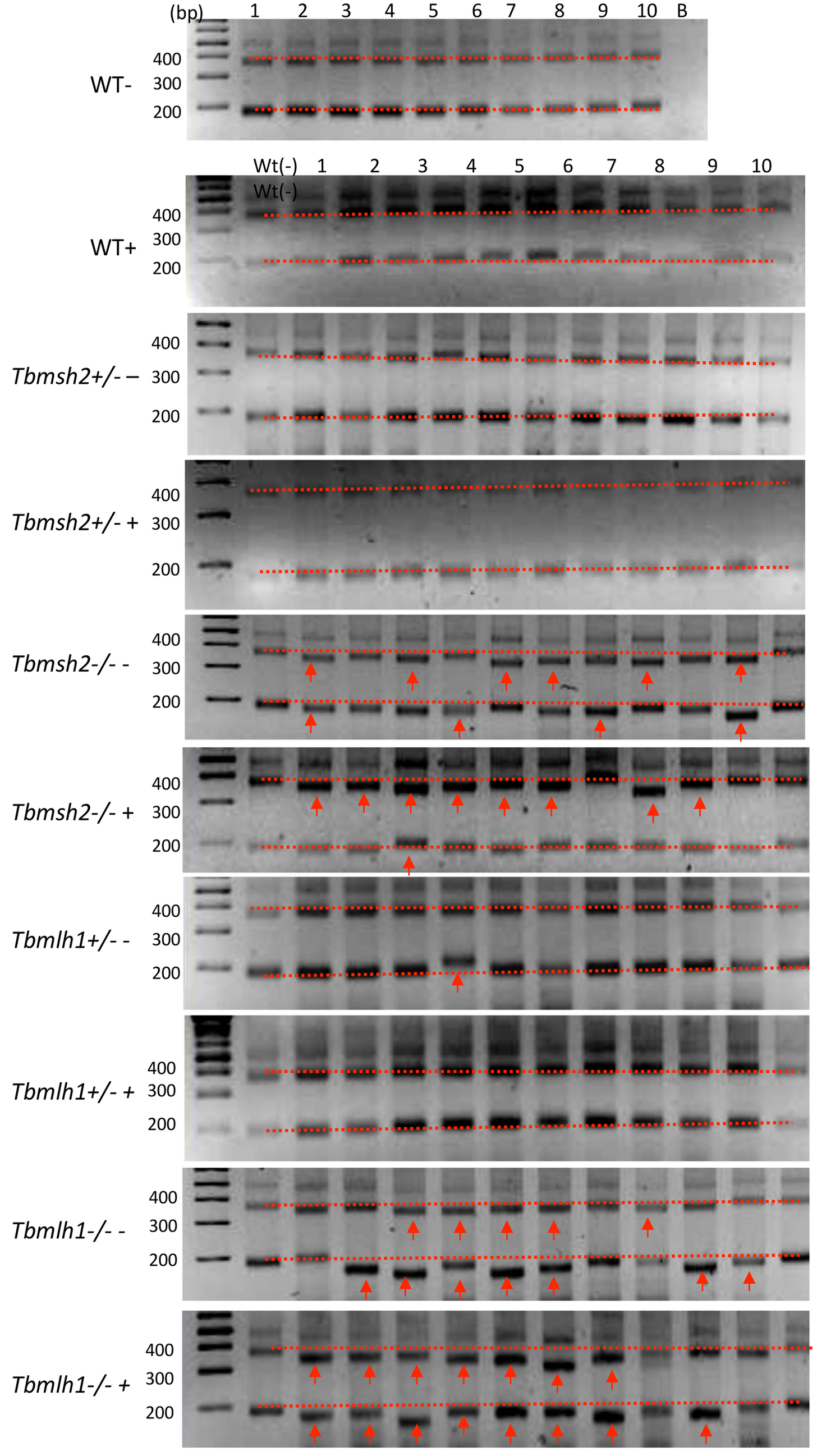

Supplement: S4 Fig — PCR amplification of the microsatellite locus JS2 from PCF WT, Tbmsh2+/-, Tbmsh2-/-, Tbmlh1+/-, and Tbmlh1-/- grown in the absence (-) and presence (+) of 20μM H2O2 for 48 hours and then cloned by limiting dilution in 96 well culture dishes. The JS2 locus was amplified from 10 clones from each cell line using primers JS2A and JS2B [21]. PCR products were separated on 3% agarose gesl; note, the two alleles of this JS2 locus have distinct sizes. PCR product from one of the wild type sub clones was also added in the first and last lane parallel to the mutants, for size comparison. Clones that show a difference in size relative to WT are indicated by an arrow; size markers are shown. (TIF) [file pntd.0003870.s004.tif]

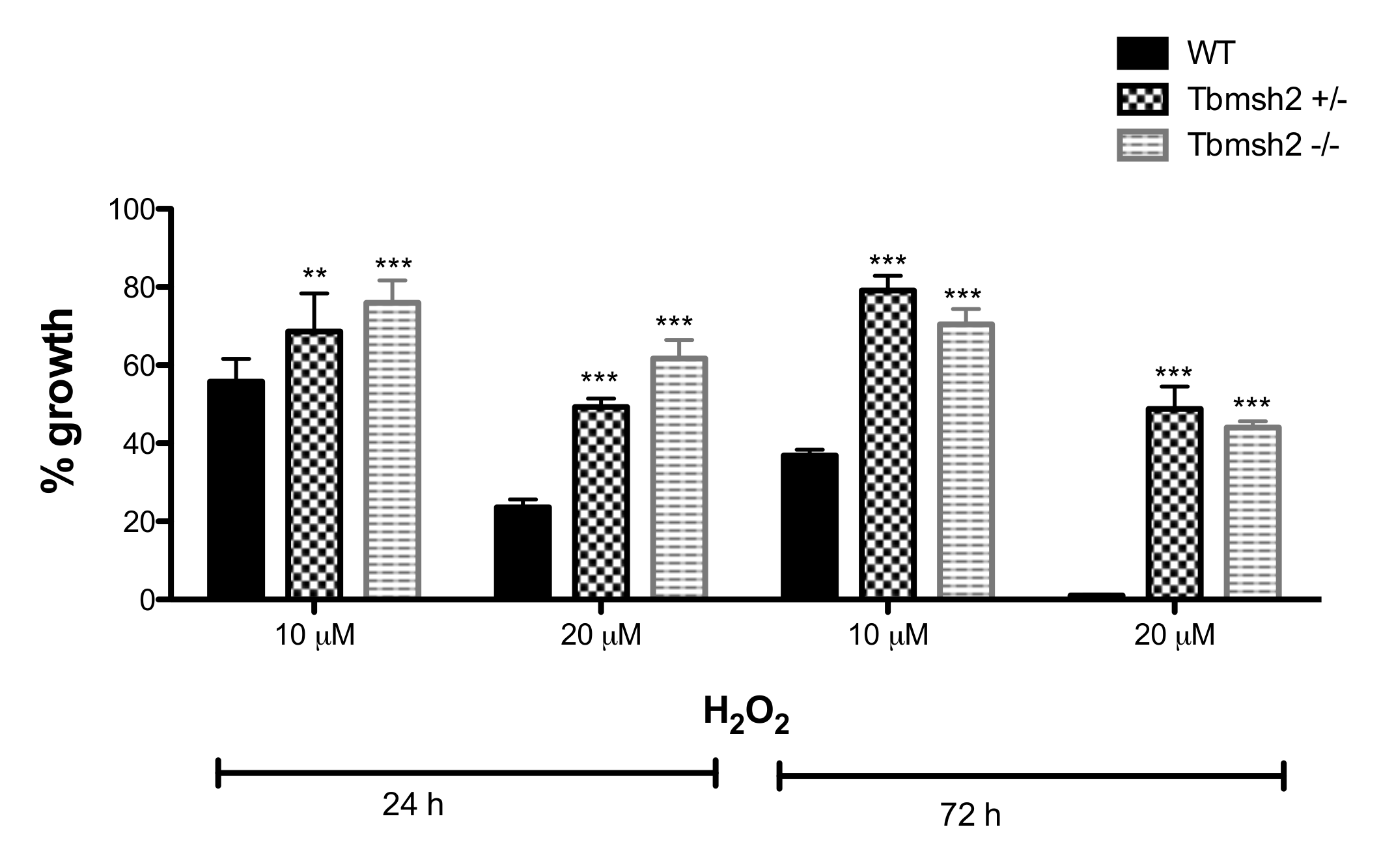

Supplement: S5 Fig — T. brucei wild type (WT), msh2+/-, msh2-/-, mlh1+/- and mlh1-/- procyclic form cells were grown in culture medium with 0 μM, 10 μM or 20 μM H2O2. Cell density was measured after 24 and 72 hours and plotted as the percentage survival of the H2O2 treated cells relative to untreated; vertical lines show standard deviation. ***p<0.001, **p<0.001, difference between mutants relative to wild type. (TIF) [file pntd.0003870.s005.tif]

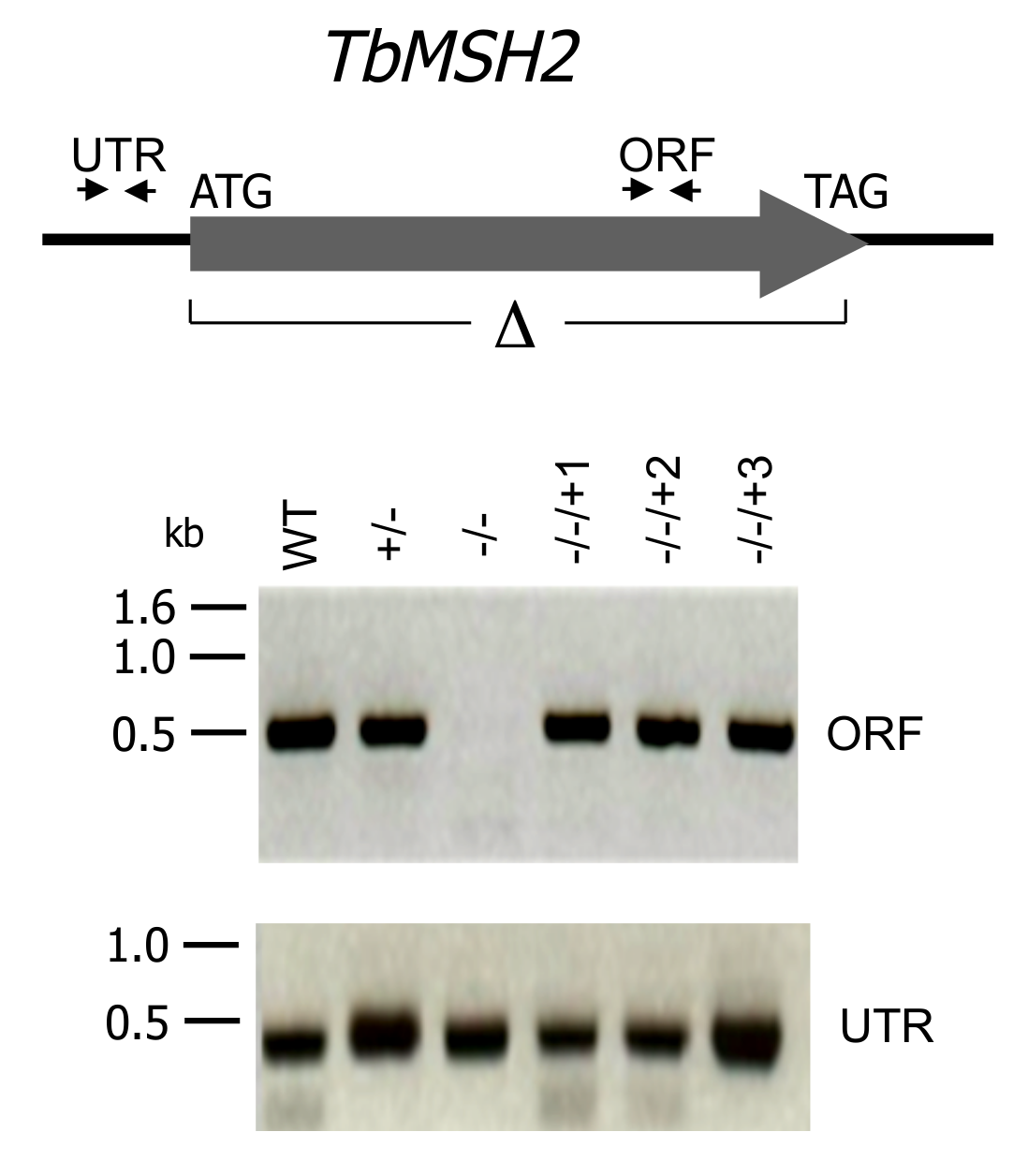

Supplement: S6 Fig — The upper diagram shows that the gene knockout strategy deletes the entire TbMSH2 ORF and indicates the position of primers (‘ORF’) used to test for the presence or absence of MSH2 ORF in genomic DNA of wild type (WT) cells, Tbmsh2+/- and Tbmsh2-/- mutants, and in three PCF Tbmsh2-/-/+ clones (1–3) in which the MSH2 ORF was reintegrated into the endogenous locus of the-/- mutant using a construct described previously [21]. The lower diagram shows two agarose gels, the uppermost displaying PCR products generated using the ORF primers and the different cells, relative to a control PCR (lower gel) in which the unaltered MSH2 5’ UTR was PCR-amplified (‘UTR’); size markers are shown (kb). (TIF) [file pntd.0003870.s006.tif]

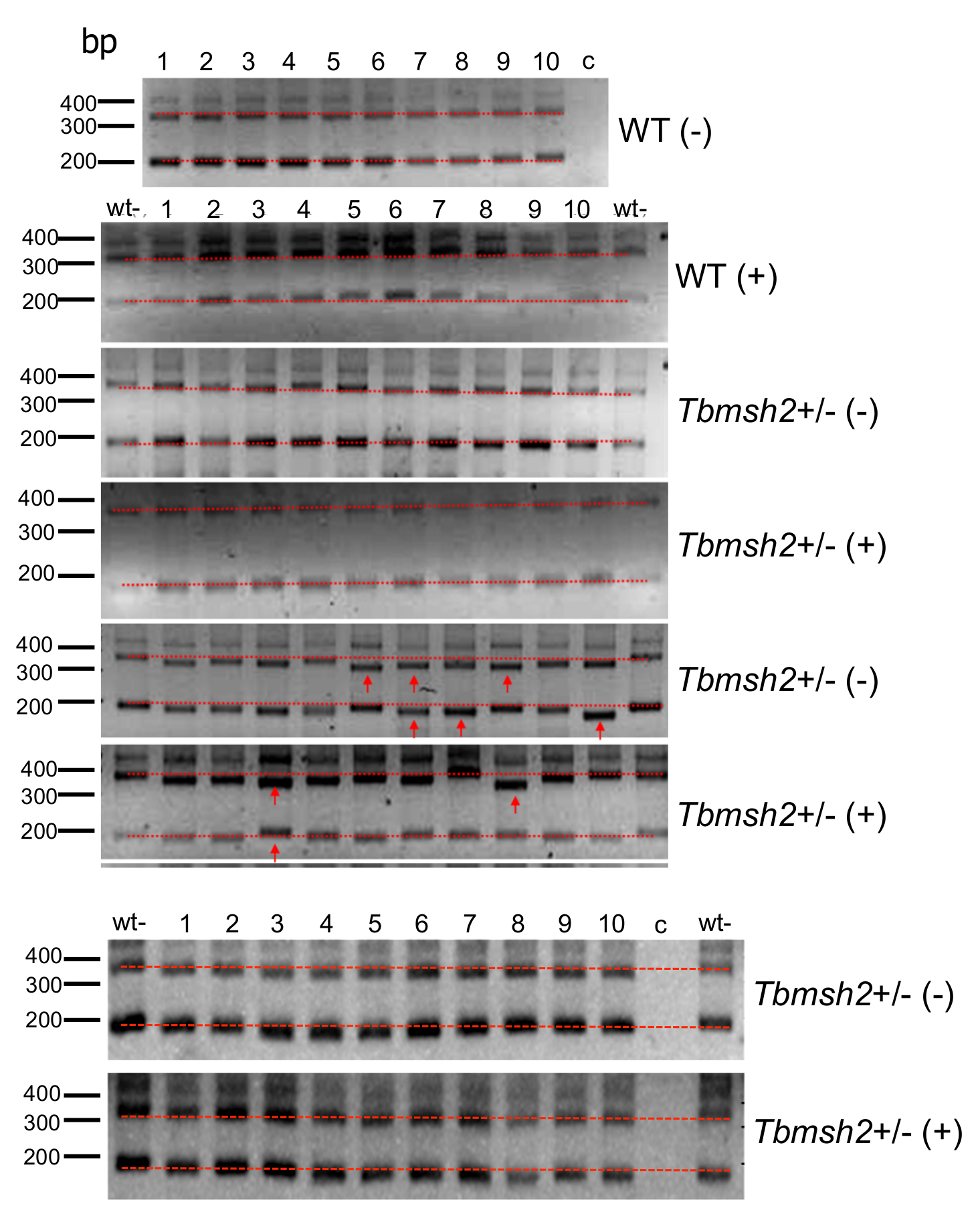

Supplement: S7 Fig — WT, Tbmsh2+/-, Tbmsh2-/- and Tbmsh2-/-/+ cells were grown in the absence (-) or presence (+) of 20 μM H2O2 for 48 hours and then cloned by limiting dilution in 96 well culture dishes. The JS2 locus was amplified from 10 clones from each cell line using primers JS2A and JS2B [21] and PCR products were separated on 3% agarose gel; note, the two alleles of the JS2 locus are distinct sizes and are marked by a dashed red line (‘c’ indicates a control PCR reaction run without genomic DNA). PCR product from one of the untreated wild type sub clones (wt-) was added in the first and last lane of the gels showing the mutants, for size comparison. Clones that show a difference in size relative to WT are indicated by an arrow; size markers are shown (bp). (TIF) [file pntd.0003870.s007.tif]

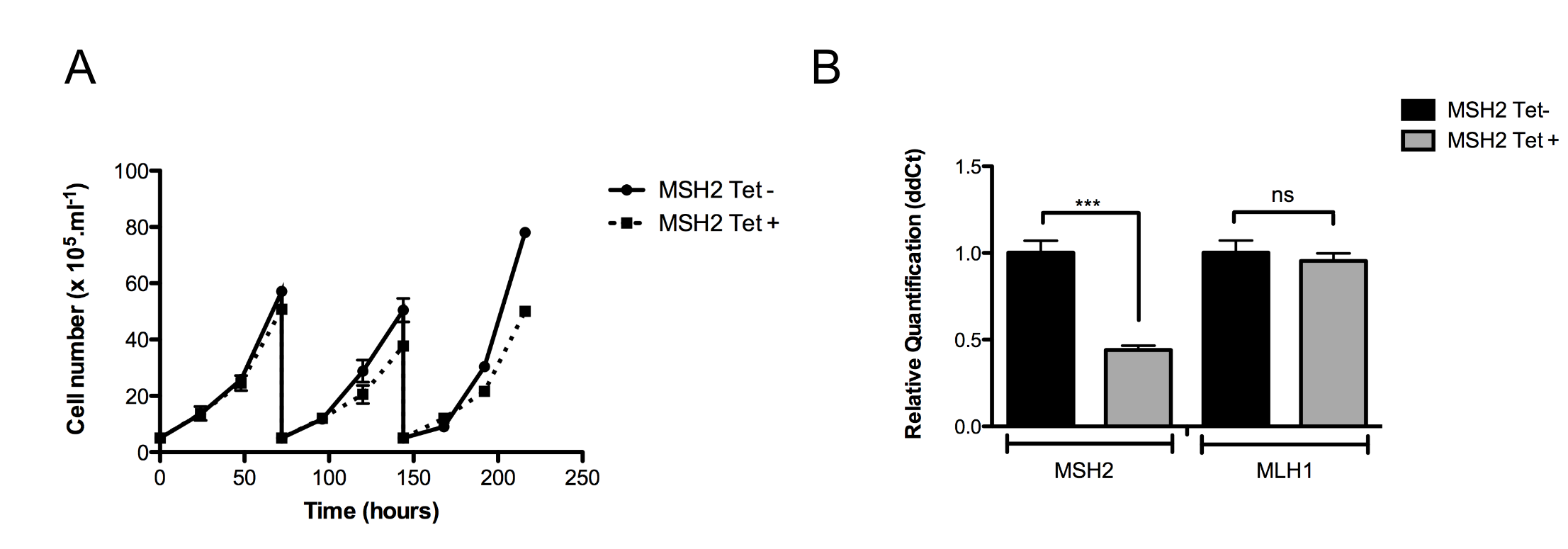

Supplement: S8 Fig — (A) Growth curve of T. brucei PCF cells in which RNAi against MSH2 was induced by the presence of tetracycline (Tet+) (2 μg.ml-1) Cell density was measured every 24 hours over a period of 216 hours and compared between the Tet+ cells and PCF cells grown in the absence of tetracycline (Tet-).; the cultures were diluted to their starting density every 72 hours. The graph shows the average cell density of two independent clones, repeated twice; vertical lines indicate standard error. P<0.001 (B) mRNA levels of MSH2 and MLH1 were determined in one cloned T. brucei PCF cell line before and after RNAi induction by quantitative reverse-transcriptase PCR. Total RNA extracted from RNAi-induced (Tet+) and non-induced (Tet-) T. brucei PCF cells was reverse transcribed and quantified using GPI8 mRNA as an endogenous control. Error bars are standard deviations for four replicates. ***P<0.001 (TIF) [file pntd.0003870.s008.tif]

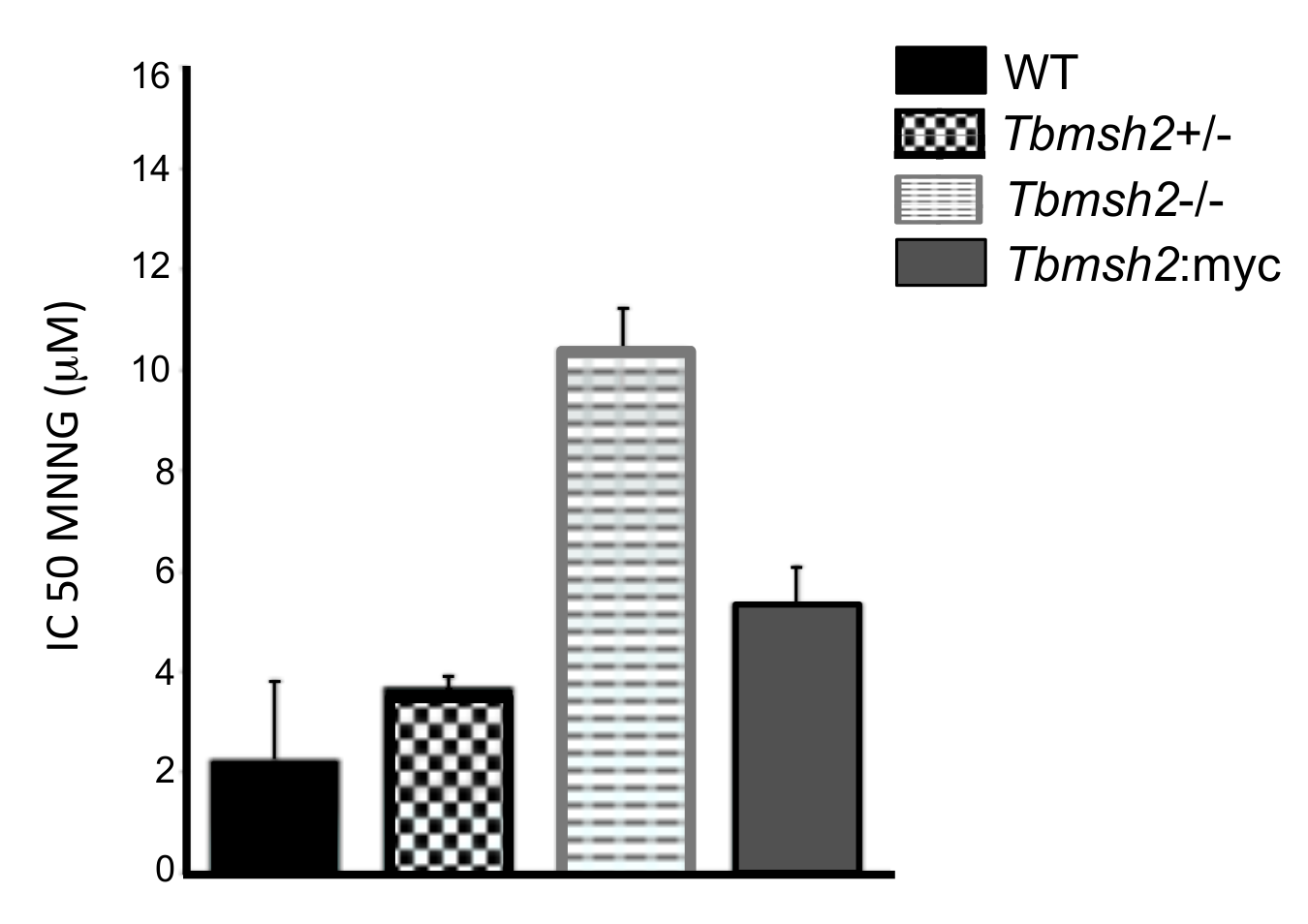

Supplement: S9 Fig — T. brucei BSF Tbmsh2+/- were transfected to express MSH2 C-terminally fused to a c-myc tag (Tbmsh2:myc) and the MMR efficiency was compared in these cells to T. brucei wild type (WT), Tbmsh2+/- and Tbmsh2-/- cells after treatment with MNNG. Cells were grown at a starting density of 1 x 10^5^ cells.ml-1 in the presence of increasing concentrations of MNNG (from 0.39 μM—400 μM) in fluorescence-readable 96 well plates. After 48 hours, Alamar Blue was added to each well and fluorescence was measured after a further 24 hours of growth. IC50 values were determined and are shown as the mean of three experiments, with standard deviations indicated by vertical bars. (TIF) [file pntd.0003870.s009.tif]
